# Trends in the Outcomes of Advanced Hepatobiliary‐Pancreatic Surgery: The Impact of a Nationwide Clinical Database and Surgeon Certification System

**DOI:** 10.1002/jhbp.12158

**Published:** 2025-05-13

**Authors:** Takayuki Anazawa, Hiroyuki Yamamoto, Etsuro Hatano, Mitsukazu Gotoh, Masafumi Nakamura, Masayuki Ohtsuka, Itaru Endo

**Affiliations:** ^1^ Division of Hepato‐Biliary‐Pancreatic Surgery and Transplantation, Department of Surgery, Graduate School of Medicine Kyoto University Kyoto Japan; ^2^ The Japanese Society of Hepato‐Biliary‐Pancreatic Surgery Tokyo Japan; ^3^ Department of Healthcare Quality Assessment, Graduate School of Medicine The University of Tokyo Tokyo Japan; ^4^ National Clinical Database Tokyo Japan

**Keywords:** hepatobiliary‐pancreatic surgery, National Clinical Database, pancreaticoduodenectomy, risk‐adjusted surgical outcome, surgeon certification system

## Abstract

**Background:**

The Japanese Society of Hepatobiliary and Pancreatic Surgery has established a certification system for experienced surgeons. Evaluating its efficacy requires accounting for patient risk variations. The National Clinical Database (NCD) facilitates this using risk‐adjusted outcome measures to validate and compare surgical performance.

**Methods:**

We analyzed the NCD from 2014 to 2020 to examine trends in adjusted odds ratios (AORs) for mortality and morbidity following pancreaticoduodenectomy using 2014 as the reference. Primary outcomes were surgical and 30‐day postoperative mortality. Secondary outcomes included severe complications and grade C pancreatic fistula. Subgroup analyses considered surgeon and institutional certification.

**Results:**

Analysis of 78 972 pancreaticoduodenectomy reports revealed a decrease in the AOR for surgical mortality from 0.906 (95% Confidence Interval [CI]: 0.759–1.082, *p* = 0.276) in 2015 to 0.647 (95% CI: 0.539–0.777, *p* < 0.001) in 2020. A significant downward trend in the incidence of Grade C pancreatic fistula was observed. Board‐certified surgeons have demonstrated superior performance compared to nonboard‐certified surgeons since 2014, with board‐certified training institutions having significantly lower AORs than those without certification. The AOR for surgical mortality showed an annual decrease across institutions.

**Conclusions:**

The certification system for hepatobiliary‐pancreatic surgery and participation in the NCD significantly decreased surgical mortality after pancreaticoduodenectomy.

## Graphical abstract

Anazawa and colleagues conducted a retrospective longitudinal cohort study using Japan's National Clinical Database to assess advanced hepatobiliary‐pancreatic surgery outcomes from 2014 to 2020. Risk‐adjusted mortality after pancreaticoduodenectomy declined, attributed to the implementation of a nationwide database and surgeon certification system, largely through fewer severe postoperative complications, particularly Grade C pancreatic fistulas.

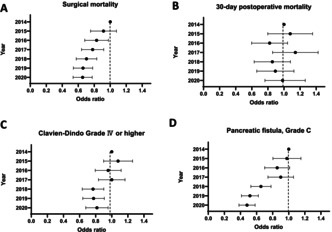

## 1 Introduction

Various initiatives have been implemented to improve surgical outcomes by reducing postoperative mortality and complications. These include enhanced surgeon training and certification [1], implementation of clinical guidelines [2], and quality improvement programs [3]. Hepatobiliary and pancreatic surgery, particularly high‐risk [4, 5], presents a significant opportunity for improvement. In 2008, the Japanese Society of Hepatobiliary and Pancreatic Surgery (JSHBPS) introduced a board certification system for expert surgeons to enhance the safety of advanced hepatobiliary and pancreatic procedures. Subsequent reports have indicated appropriate surgeries at certified training institutions and a decline in mortality rates for complex hepatobiliary and pancreatic surgeries [1, 6]. Although a structural framework has been established to ensure quality medical care, assessing its impact on outcomes requires more than comparing case numbers and mortality rates from registered data. A comprehensive evaluation must consider the varying risk profiles of individual patients.

National quality improvement initiatives such as the American College of Surgeons National Surgical Quality Improvement Program (ACS NSQIP) and Japan's National Clinical Database (NCD) have demonstrated significant reductions in postoperative morbidity and mortality rates [7, 8, 9]. These nationwide efforts, characterized by comprehensive data collection, risk‐adjusted analysis, and targeted quality improvement strategies, have led to positive changes in surgical care delivery. Such collective endeavors underscore the critical importance of collaborative approaches in enhancing postoperative outcomes [10].

Since its inception in January 2011, the NCD has developed a risk model for major surgical procedures, based on systematically collected large‐scale data [4, 5]. This model enables benchmarking by comparing the surgical performance (mortality and morbidity) adjusted for patient severity using risk‐adjusted outcomes. Analyzing performance based on these outcomes over time can highlight the strengths and areas for improvement in hepatobiliary surgery. The American College of Surgeons National Surgical Quality Improvement Program (ACS NSQIP), similar to the NCD, evaluated temporal changes in the observed‐to‐expected ratio of postoperative mortality and complication rates at participating centers [11]. Although the conclusion that participation in the American College of Surgeons National Surgical Quality Improvement Program (ACS‐NSQIP) is directly responsible for improved outcomes is disputed, given that postoperative outcomes tend to improve even in non‐participating centers [12, 13], subsequent reports indicate that participation in the ACS‐NSQIP can enhance postoperative outcomes [9].

In Japan, the widespread participation of healthcare facilities in the NCD precludes comparisons based on participation. However, the board certification system for hepatobiliary‐pancreatic surgery specialists incorporates criteria for training institutions, including case volume and certified instructors [6], allowing comparisons based on certification status. This study aimed to evaluate how the outcomes of pancreaticoduodenectomy, a frequently performed advanced hepatobiliary‐pancreatic surgery, have evolved over time using risk‐adjusted outcome measures, and to determine if improvements in surgical outcomes persist even after accounting for patient backgrounds, surgeon certification status, and institutional training status, and to identify potential areas for enhancing surgical performance.

## 2 Methods

### 2.1 Study Design, Database and Outcomes

This study used retrospective longitudinal cohort analysis of the NCD dataset. The research protocol for this study was approved by the Ethics Committee of the Graduate School and Faculty of Medicine of Kyoto University (approval code: R3680). The NCD, developed in conjunction with Japan's board certification system, is a prospective clinical registry designed to provide risk‐adjusted outcome feedback to hospitals and surgeons for quality improvement purposes. By 2021, the NCD encompasses over 1 500 000 surgical cases annually, with participation from approximately 5400 institutions by 2021 [14]. Data quality is maintained through audits of the selected facilities. The database facilitates the calculation of the observed and expected surgical mortality and morbidity using the established risk models. We extracted data on patients who underwent pancreaticoduodenectomy, which had been previously established in the risk model [4, 15], from 2014 to 2020 across all NCD‐participating institutions. Excluding laparoscopic and robotic procedures, we analyzed the temporal trend of risk‐adjusted odds ratios for mortality and morbidity following pancreaticoduodenectomy, using 2014 as the reference year. The primary outcome was surgical mortality, defined as death within 30 days after surgery or during hospitalization (up to 90 days after surgery). Secondary outcomes included 30‐day postoperative mortality, Clavien‐Dindo grade IV or higher complication rates, and grade C pancreatic fistula [16] as defined by the International Study Group of Pancreatic Fistula (ISGPF) [17]. Surgical mortality was defined as death within 30 days (regardless of hospitalization) or death during hospitalization. This outcome was used in several surgical database studies [3].

### 2.2 Board Certification System and Monitoring the Safety of Advanced Hepatobiliary and Pancreatic Surgery

The Japan Society of Hepato‐Biliary‐Pancreatic Surgery (JSHBPS) previously outlined its comprehensive system for certifying expert surgeons, with application requirements detailed in Table S1 [6]. Advanced hepatobiliary and pancreatic surgery includes anatomical hepatectomy, liver transplantation for both donors and recipients, and complex pancreatic operations such as pancreaticoduodenectomy. Certification is contingent upon JSHBPS's approval of the applicant's surgical proficiency. The certification examination pass rates are 46.7%, 52.5%, and 36.8% in 2021, 2022, and 2023, respectively, with more than 600 surgeons having attained certification to date.

The JSHBPS Safety Management Committee conducted analyses of summary reports detailing surgical volumes and the associated 30‐day and 90‐day mortality rates. These findings were disseminated at the Board of Directors' meetings and JSHBPS annual conferences, with subsequent feedback provided to training institutions. Institutions showing a 90‐day mortality rate of 5% or higher over a three‐year period are required to submit comprehensive reports encompassing preoperative risk factors, surgical specifics, clinical progression until death, and cause of death. The Safety Management Committee evaluates these reports and provides recommendations to the Board of Directors, which determines whether the institution requires consultation or guidance regarding surgical safety practices. When deemed necessary, guidance was facilitated through on‐site evaluations conducted by certified Safety Management Committee members.

Subgroup analyses were performed considering whether the primary surgeon was board‐certified in hepatobiliary and pancreatic surgery, as well as the training status of the institution. Board‐certified institutions were categorized as “A” (≥ 50 advanced hepatobiliary and pancreatic surgeries annually) or “B” (30–49 such surgeries annually), with non‐certified institutions forming the third category. If institutions do not meet the accreditation requirements, they become nonboard‐certified training institutions.

### 2.3 Statistical Analysis

We used a multivariable logistic regression model to evaluate the temporal changes in surgical outcomes using a risk‐adjusted measure for pancreaticoduodenectomy. The analysis incorporated surgical mortality, 30‐day postoperative mortality, severe complications (Clavien–Dindo grade IV or higher), and grade C pancreatic fistula as dependent variables and the following independent variables that were found to contribute to effective risk model development in previous reports [4, 15]: year of surgery, age, sex, chronic obstructive pulmonary disorder (COPD), bleeding disorder, American Society of Anesthesiologists (ASA) class, preoperative activities of daily life (ADL), body mass index (BMI), weight loss, Brinkman index, respiratory distress, angina, myocardial infarction, arterial occlusive disease, cerebrovascular disease, ascites, multiple metastatic tumors, tumor type, emergency operation, intraoperative blood loss, operation time, vascular reconstruction, as well as the following laboratory results: WBC, hemoglobin, hematocrit, platelet count, urea nitrogen, creatinine, albumin, serum Na, PT‐INR, and APTT. The results are expressed as adjusted odds ratios (AORs) with corresponding 95% confidence intervals (CIs).

Furthermore, we used a multivariate logistic regression model with generalized estimating equations to account for clustering at the surgeon and institutional levels. An exchangeable correlation matrix structure was also used. The analysis incorporated surgical mortality, 30‐day postoperative mortality, Clavien‐Dindo grade IV or higher complication rates, and grade C pancreatic fistula as the dependent variables. The independent variables included the year of surgery, established risk model variables for each outcome, and an interaction term between the year of surgery and the surgeon‐ or institution‐level category.

All statistical analyses were conducted using the STATA 17 software (Stata, College Station, Texas, USA).

## 3 Results

### 3.1 Patient Characteristics and Crude Surgical Mortality and Morbidity

Between 10 000 and 12 000 pancreaticoduodenectomies were performed annually, with a total of 78 972 cases reported to the NCD in the study cohort. The detailed patient characteristics are presented in Table 1. The proportion of elderly patients, a known risk factor for surgical mortality [4], has increased over time; the percentage of patients over 80 years of age increased from 11.8% in 2014 to 16.0% in 2020. Among the previously identified risk factors [4], the proportion of patients with ASA class 3 or higher, Brinkman index > 400, and BMI > 25 kg/m^2^ demonstrated an annual upward trend. The median intraoperative estimated blood loss decreased from 735 mL in 2014 to 560 mL in 2020, whereas the median operation time remained stable. Surgical mortality rates for 2014–2020 were 2.6%, 2.3%, 2.2%, 2.1%, 1.9%, 1.8%, and 1.9%, respectively. The corresponding 30‐day mortality rates were 1.1%, 1.1%, 0.9%, 1.3%, 1.0%, 1.0%, and 1.2%. Trends in Clavien‐Dindo grade IV or higher complications and grade C pancreatic fistula incidence are also presented in Table 1, with rates decreasing from 2.7% and 2.4% in 2014 to 2.3% and 1.2% in 2020, respectively.

**TABLE 1:** Patient characteristics.

	2014	2015	2016	2017	2018	2019	2020
	*N* = 10 400	*N* = 10 577	*N* = 11 025	*N* = 11 582	*N* = 11 625	*N* = 11 812	*N* = 11 951
Age (Years)							
−59	1454 (14.0%)	1506 (14.2%)	1564 (14.2%)	1594 (13.8%)	1549 (13.3%)	1553 (13.1%)	1544 (12.9%)
60–64	1313 (12.6%)	1235 (11.7%)	1140 (10.3%)	1135 (9.8%)	1057 (9.1%)	1074 (9.1%)	1016 (8.5%)
65–69	2042 (19.6%)	2112 (20.0%)	2271 (20.6%)	2359 (20.4%)	2191 (18.8%)	2053 (17.4%)	1837 (15.4%)
70–74	2347 (22.6%)	2419 (22.9%)	2403 (21.8%)	2414 (20.8%)	2579 (22.2%)	2673 (22.6%)	2877 (24.1%)
75–79	2014 (19.4%)	2039 (19.3%)	2243 (20.3%)	2502 (21.6%)	2557 (22.0%)	2612 (22.1%)	2763 (23.1%)
80+	1230 (11.8%)	1266 (12.0%)	1404 (12.7%)	1578 (13.6%)	1692 (14.6%)	1847 (15.6%)	1914 (16.0%)
Male	6337 (60.9%)	6423 (60.7%)	6741 (61.1%)	7074 (61.1%)	7014 (60.3%)	7170 (60.7%)	7239 (60.6%)
COPD	362 (3.5%)	384 (3.6%)	443 (4.0%)	416 (3.6%)	368 (3.2%)	415 (3.5%)	403 (3.4%)
Bleeding disorder	376 (3.6%)	411 (3.9%)	417 (3.8%)	511 (4.4%)	338 (2.9%)	344 (2.9%)	386 (3.2%)
ASA class (grade 3, 4, and 5)	1157 (11.1%)	1224 (11.6%)	1429 (13.0%)	1459 (12.6%)	1621 (13.9%)	1716 (14.5%)	1869 (15.6%)
ASA class (grade 4 and 5)	23 (0.2%)	32 (0.3%)	34 (0.3%)	25 (0.2%)	34 (0.3%)	26 (0.2%)	38 (0.3%)
ADL within 30 days before surgery (Partially/totally dependent)	268 (2.6%)	251 (2.4%)	278 (2.5%)	243 (2.1%)	226 (1.9%)	224 (1.9%)	234 (2.0%)
BMI > 25	1678 (16.1%)	1712 (16.2%)	1796 (16.3%)	1998 (17.3%)	2025 (17.4%)	2161 (18.3%)	2194 (18.4%)
Weight loss > 10%	627 (6.0%)	624 (5.9%)	598 (5.4%)	598 (5.2%)	572 (4.9%)	536 (4.5%)	558 (4.7%)
Brinkman index > 400	3183 (30.6%)	3371 (31.9%)	3700 (33.6%)	3833 (33.1%)	3875 (33.3%)	4125 (34.9%)	4229 (35.4%)
Brinkman index > 600	2493 (24.0%)	2595 (24.5%)	2834 (25.7%)	2942 (25.4%)	2949 (25.4%)	3120 (26.4%)	3191 (26.7%)
Respiratory distress (within 30 days before surgery)	88 (0.8%)	92 (0.9%)	112 (1.0%)	97 (0.8%)	69 (0.6%)	102 (0.9%)	110 (0.9%)
Angina (within 30 days before surgery)	114 (1.1%)	127 (1.2%)	125 (1.1%)	131 (1.1%)	128 (1.1%)	106 (0.9%)	128 (1.1%)
Myocardial infarction (within 6 months before surgery)	34 (0.3%)	36 (0.3%)	31 (0.3%)	36 (0.3%)	45 (0.4%)	28 (0.2%)	40 (0.3%)
Arterial occlusive disease	49 (0.5%)	31 (0.3%)	43 (0.4%)	37 (0.3%)	43 (0.4%)	56 (0.5%)	38 (0.3%)
Previous cerebrovascular disease	321 (3.1%)	281 (2.7%)	296 (2.7%)	416 (3.6%)	465 (4.0%)	543 (4.6%)	504 (4.2%)
Ascites without control	107 (1.0%)	126 (1.2%)	123 (1.1%)	114 (1.0%)	112 (1.0%)	102 (0.9%)	118 (1.0%)
WBC count > 11 000/μL	208 (2.0%)	222 (2.1%)	227 (2.1%)	235 (2.0%)	247 (2.1%)	226 (1.9%)	278 (2.3%)
Hemoglobin levels < 7 g/dL	33 (0.3%)	20 (0.2%)	21 (0.2%)	31 (0.3%)	32 (0.3%)	23 (0.2%)	28 (0.2%)
Hematocrit (> 48%, male > 42%, female)	107 (1.0%)	139 (1.3%)	159 (1.4%)	180 (1.6%)	186 (1.6%)	194 (1.6%)	216 (1.8%)
Platelet count < 80 000/μL	43 (0.4%)	35 (0.3%)	44 (0.4%)	51 (0.4%)	44 (0.4%)	48 (0.4%)	49 (0.4%)
Platelet count < 120 000/μL	288 (2.8%)	327 (3.1%)	308 (2.8%)	334 (2.9%)	303 (2.6%)	322 (2.7%)	322 (2.7%)
Serum urea nitrogen levels < 8 mg/dL	527 (5.1%)	553 (5.2%)	608 (5.5%)	565 (4.9%)	543 (4.7%)	542 (4.6%)	535 (4.5%)
Serum creatinine levels > 2 mg/dL	119 (1.1%)	107 (1.0%)	116 (1.1%)	127 (1.1%)	144 (1.2%)	140 (1.2%)	154 (1.3%)
Serum creatinine levels > 3 mg/dL	82 (0.8%)	69 (0.7%)	81 (0.7%)	87 (0.8%)	97 (0.8%)	91 (0.8%)	112 (0.9%)
Serum albumin levels < 2.5 g/dL	209 (2.0%)	189 (1.8%)	217 (2.0%)	263 (2.3%)	235 (2.0%)	243 (2.1%)	219 (1.8%)
Serum sodium level > 146 mEq/L	45 (0.4%)	34 (0.3%)	28 (0.3%)	28 (0.2%)	45 (0.4%)	45 (0.4%)	55 (0.5%)
Serum CRP levels > 1.0 mg/dL	1742 (16.8%)	1745 (16.5%)	1693 (15.4%)	1929 (16.7%)	1805 (15.5%)	1848 (15.6%)	1840 (15.4%)
PT‐INR > 1.1	1162 (11.2%)	1115 (10.5%)	1199 (10.9%)	1154 (10.0%)	942 (8.1%)	935 (7.9%)	1000 (8.4%)
PT‐INR > 1.25	349 (3.4%)	347 (3.3%)	285 (2.6%)	331 (2.9%)	303 (2.6%)	281 (2.4%)	304 (2.5%)
APTT > 40 s	403 (3.9%)	442 (4.2%)	530 (4.8%)	450 (3.9%)	389 (3.3%)	341 (2.9%)	338 (2.8%)
Duodenal cancer	344 (3.3%)	381 (3.6%)	362 (3.3%)	399 (3.4%)	387 (3.3%)	402 (3.4%)	400 (3.3%)
Perihilar bile duct carcinoma	261 (2.5%)	248 (2.3%)	261 (2.4%)	241 (2.1%)	211 (1.8%)	188 (1.6%)	154 (1.3%)
Extrahepatic bile duct carcinoma	2090 (20.1%)	2083 (19.7%)	2175 (19.7%)	2310 (19.9%)	2217 (19.1%)	2200 (18.6%)	2242 (18.8%)
Gallbladder cancer	104 (1.0%)	97 (0.9%)	93 (0.8%)	110 (0.9%)	94 (0.8%)	79 (0.7%)	92 (0.8%)
Ampulla of Vater carcinoma	1227 (11.8%)	1223 (11.6%)	1249 (11.3%)	1275 (11.0%)	1225 (10.5%)	1256 (10.6%)	1233 (10.3%)
Multiple metastatic tumor	41 (0.4%)	40 (0.4%)	46 (0.4%)	57 (0.5%)	36 (0.3%)	31 (0.3%)	38 (0.3%)
Emergency operation	79 (0.8%)	83 (0.8%)	64 (0.6%)	70 (0.6%)	87 (0.7%)	65 (0.6%)	70 (0.6%)
Intraoperative estimated blood loss (mL)							
Median (IQR)	735 (434–1211.5)	716 (420–1200)	690 (400–1135)	650 (375–1089)	600 (340–1004)	570 (315–980)	560 (310–957)
Operation time (min)							
Median (IQR)	457 (378–545)	456 (377–544)	460 (383–549)	454 (376–544)	455 (377–544)	453 (375–539)	453 (376–539)
Vascular reconstruction	1291 (12.4%)	1332 (12.6%)	1429 (13.0%)	1341 (11.6%)	1427 (12.3%)	1367 (11.6%)	1581 (13.2%)
Length of hospital stay (days)							
Median (IQR)	29 (20–42)	28 (19–41)	28 (19–40)	27 (19–40)	27 (18–39)	26 (18–38)	25 (18–37)
Observed surgical mortality	267 (2.6%)	247 (2.3%)	241 (2.2%)	240 (2.1%)	218 (1.9%)	215 (1.8%)	222 (1.9%)
30‐day mortality	111 (1.1%)	120 (1.1%)	98 (0.9%)	145 (1.3%)	111 (1.0%)	119 (1.0%)	138 (1.2%)
Clavien‐dindo grade IV or higher	278 (2.7%)	301 (2.8%)	284 (2.6%)	318 (2.7%)	248 (2.1%)	258 (2.2%)	280 (2.3%)
Pancreatic fistula, grade C	253 (2.4%)	246 (2.3%)	230 (2.1%)	258 (2.2%)	187 (1.6%)	152 (1.3%)	143 (1.2%)

Abbreviations: ADL, activity of daily life; APTT, activated partial thromboplastin time; ASA, American Society of Anesthesiologists; BMI, body mass index; COPD, chronic obstructive pulmonary disorder; CRP, C reactive protein; PT‐INR, prothrombin time‐international normalized ratio; WBC, white blood cell.

### 3.2 Annual Change About Primary and Secondary Outcomes

**FIGURE 1:**
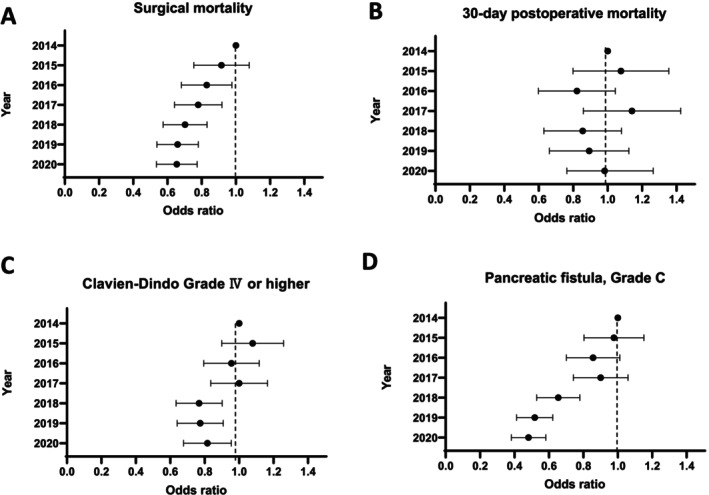
Annual change in odds ratios for surgical mortality (A), 30‐day postoperative mortality (B), Clavien–Dindo ≥ Grade IV (C), and Pancreatic fistula, Grade C (D).

Figure 1A–D illustrate the annual changes in AOR for surgical mortality (A), 30‐day postoperative mortality (B), Clavien‐Dindo grade IV or higher complications (C), and pancreatic fistula grade C (D). The AOR with the corresponding 95% CI for surgical mortality from 2014 to 2020 demonstrated a clear year‐on‐year downward trend. This trend indicates an improved performance in the primary endpoint of surgical mortality. Table 2 presents the AOR values for the year 2014. The AOR was 0.906 (95% CI: 0.759–1.082, *p* = 0.276) in 2015 and significantly decreased to 0.647 (95% CI: 0.539–0.777, *p* < 0.001) in 2020.

**TABLE 2:** Time trend in the adjusted odds ratios.

Year	All cases				Nonboard certificated surgeon				Board certificated surgeon				Nonboard‐certified training institutions				Board‐certified A training institutions				Board‐certified B training institutions			
	AOR	95% CI		*p*	AOR	95% CI		*p*	AOR	95% CI		*p*	AOR	95% CI		*p*	AOR	95% CI		*p*	AOR	95% CI		*p*
		min	max			min	max			min	max			min	max			min	max			min	max	
Surgical mortality																								
2014	Ref*	Ref*	Ref*		Ref**	Ref**	Ref**		0.817	0.603	1.108	0.193	Ref***	Ref***	Ref***		0.434	0.32	0.587	< 0.001	0.599	0.38	0.947	0.028
2015	0.906	0.759	1.082	0.276	0.925	0.75	1.141	0.467	0.724	0.547	0.959	0.024	0.899	0.725	1.114	0.33	0.404	0.298	0.547	< 0.001	0.602	0.4	0.906	0.015
2016	0.82	0.686	0.981	0.03	0.798	0.649	0.98	0.031	0.761	0.577	1.004	0.054	0.803	0.648	0.994	0.044	0.354	0.258	0.485	< 0.001	0.653	0.445	0.96	0.03
2017	0.771	0.645	0.922	0.004	0.764	0.62	0.941	0.011	0.694	0.529	0.911	0.008	0.774	0.621	0.964	0.022	0.382	0.282	0.516	< 0.001	0.451	0.303	0.673	< 0.001
2018	0.695	0.579	0.835	< 0.001	0.696	0.563	0.861	0.001	0.627	0.471	0.835	0.001	0.711	0.567	0.89	0.003	0.314	0.224	0.44	< 0.001	0.511	0.352	0.742	< 0.001
2019	0.652	0.542	0.784	< 0.001	0.66	0.533	0.818	< 0.001	0.572	0.427	0.766	< 0.001	0.791	0.631	0.993	0.044	0.262	0.19	0.363	< 0.001	0.331	0.232	0.474	< 0.001
2020	0.647	0.539	0.777	< 0.001	0.67	0.542	0.829	< 0.001	0.522	0.39	0.7	< 0.001	0.695	0.543	0.888	0.004	0.304	0.221	0.416	< 0.001	0.436	0.304	0.626	< 0.001
30 day postoperative mortality																								
2014	Ref*	Ref*	Ref*		Ref**	Ref**	Ref**		0.785	0.48	1.285	0.336	Ref***	Ref***	Ref***		0.417	0.249	0.697	0.001	0.808	0.425	1.537	0.516
2015	1.053	0.811	1.366	0.7	1.071	0.791	1.45	0.656	0.795	0.535	1.183	0.258	1.135	0.827	1.56	0.433	0.459	0.294	0.717	0.001	0.568	0.315	1.022	0.059
2016	0.801	0.609	1.054	0.113	0.761	0.55	1.054	0.1	0.741	0.489	1.121	0.156	0.821	0.588	1.146	0.247	0.308	0.188	0.502	< 0.001	0.749	0.418	1.341	0.331
2017	1.118	0.871	1.435	0.381	1.082	0.809	1.449	0.595	0.979	0.675	1.419	0.91	1.149	0.84	1.571	0.385	0.529	0.342	0.818	0.004	0.776	0.469	1.282	0.322
2018	0.835	0.64	1.089	0.184	0.835	0.612	1.138	0.253	0.699	0.456	1.073	0.101	0.95	0.672	1.342	0.77	0.317	0.192	0.524	< 0.001	0.65	0.392	1.08	0.096
2019	0.872	0.672	1.133	0.305	0.865	0.64	1.168	0.343	0.753	0.499	1.136	0.176	1.084	0.784	1.501	0.625	0.357	0.227	0.563	< 0.001	0.48	0.298	0.773	0.003
2020	0.982	0.763	1.264	0.888	1.023	0.765	1.369	0.877	0.713	0.478	1.065	0.099	1.159	0.833	1.614	0.382	0.418	0.273	0.64	< 0.001	0.653	0.423	1.008	0.054
Clavien‐dindo grade IV or higher																								
2014	Ref*	Ref*	Ref*		Ref**	Ref**	Ref**		1.137	0.87	1.486	0.347	Ref***	Ref***	Ref***		0.736	0.564	0.961	0.024	0.755	0.478	1.191	0.227
2015	1.069	0.904	1.263	0.436	1.154	0.948	1.405	0.154	1.02	0.795	1.31	0.875	1.049	0.849	1.295	0.66	0.789	0.596	1.045	0.098	0.919	0.653	1.295	0.631
2016	0.946	0.799	1.121	0.52	1.001	0.818	1.225	0.991	0.964	0.743	1.251	0.783	1.004	0.803	1.256	0.97	0.594	0.445	0.794	< 0.001	0.862	0.604	1.229	0.412
2017	0.991	0.84	1.169	0.915	1	0.819	1.22	0.996	1.126	0.882	1.438	0.34	0.905	0.719	1.137	0.391	0.837	0.644	1.089	0.185	0.776	0.538	1.122	0.177
2018	0.76	0.638	0.905	0.002	0.785	0.636	0.968	0.024	0.839	0.644	1.094	0.194	0.764	0.6	0.973	0.029	0.514	0.382	0.692	< 0.001	0.78	0.559	1.089	0.145
2019	0.766	0.644	0.911	0.003	0.801	0.653	0.983	0.034	0.827	0.635	1.077	0.158	0.883	0.701	1.113	0.291	0.484	0.355	0.662	< 0.001	0.621	0.466	0.828	0.001
2020	0.808	0.681	0.958	0.014	0.879	0.717	1.079	0.219	0.798	0.61	1.043	0.099	0.879	0.685	1.128	0.311	0.556	0.413	0.749	< 0.001	0.681	0.502	0.923	0.013
Pancreatic fistula, grade C																								
2014	Ref*	Ref*	Ref*		Ref**	Ref**	Ref**		1.245	0.938	1.653	0.13	Ref***	Ref***	Ref***		0.995	0.748	1.322	0.971	1.008	0.654	1.552	0.973
2015	0.967	0.809	1.157	0.715	1.013	0.805	1.273	0.914	1.098	0.825	1.461	0.521	0.961	0.732	1.262	0.777	0.897	0.656	1.227	0.497	1.194	0.789	1.807	0.402
2016	0.847	0.706	1.016	0.074	0.906	0.723	1.137	0.394	0.943	0.701	1.268	0.698	0.87	0.664	1.139	0.311	0.773	0.579	1.032	0.081	1.025	0.687	1.529	0.903
2017	0.891	0.747	1.064	0.203	0.963	0.77	1.206	0.745	0.965	0.718	1.298	0.816	0.939	0.719	1.226	0.645	0.845	0.615	1.161	0.3	0.906	0.596	1.376	0.643
2018	0.646	0.533	0.783	< 0.001	0.707	0.554	0.902	0.005	0.692	0.489	0.979	0.038	0.717	0.534	0.963	0.027	0.533	0.379	0.748	< 0.001	0.783	0.514	1.193	0.255
2019	0.51	0.416	0.626	< 0.001	0.569	0.441	0.734	< 0.001	0.528	0.374	0.746	< 0.001	0.595	0.441	0.804	0.001	0.468	0.328	0.668	< 0.001	0.454	0.282	0.73	0.001
2020	0.475	0.385	0.585	< 0.001	0.535	0.414	0.69	< 0.001	0.475	0.336	0.671	< 0.001	0.682	0.509	0.915	0.011	0.287	0.197	0.417	< 0.001	0.494	0.323	0.756	0.001

Abbreviation: AOR, adjusted odds ratio.

* Reference for all cases.

** Reference for nonboard and board certificated surgeon.

*** Reference for nonboard‐certificated, board certificated A, and board certificated B training institutions.

Notably, the trend in the incidence of grade C pancreatic fistula was noteworthy. The AOR with corresponding 95% CIs for pancreatic fistula incidence from 2014 to 2020 revealed a clear downward trend. As shown in Table 2, the AOR in 2015 was 0.967 (95% CI: 0.809–1.157, *p* = 0.715), which significantly decreased to 0.475 (95% CI: 0.385–0.585, *p* < 0.001) in 2020.

Clavien‐Dindo grade IV or higher complication rates showed a trend of improvement, with significant enhancements observed since 2018 (Figure 1D and Table 2); it was 1.069 (95% CI: 0.904–1.263, *p* = 0.436) in 2015 and significantly decreased to 0.808 (95% CI: 0.681–0.958, *p* = 0.014) in 2020. In contrast, the 30‐day postoperative mortality rate showed no substantial improvement over time (Figure 1B and Table 2), with values of 1.053 (95% CI: 0.811–1.366, *p* = 0.7) in 2015 and 0.982 (95% CI: 0.763–1.264, *p* = 0.888) in 2020.

### 3.3 Annual Change in Outcomes by Surgeon Certificated Status

**FIGURE 2:**
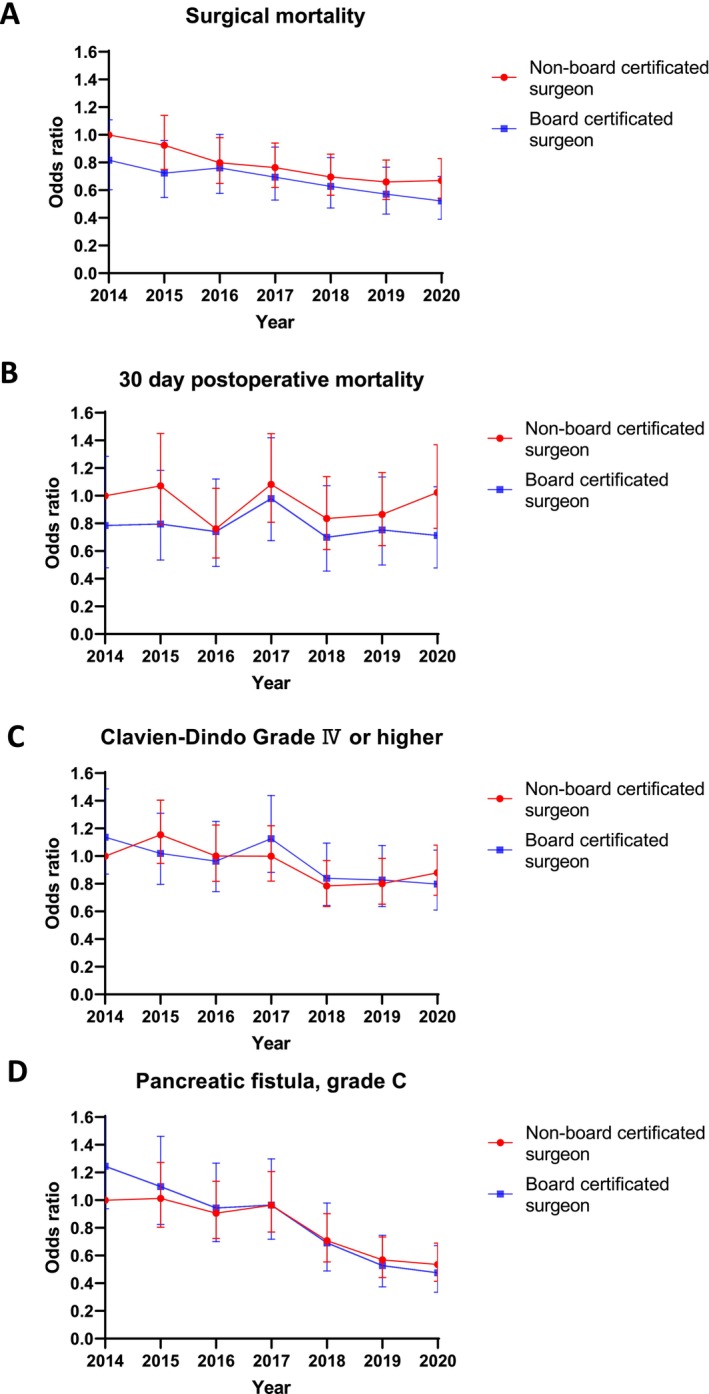
Annual change in odds ratios for surgical mortality by surgeon status. (A), 30‐day postoperative mortality (B), Clavien–Dindo ≥ Grade IV(C), and Pancreatic fistula, Grade C (D).

The AOR was then examined separately to determine whether the surgeon was a board certificated surgeon or a nonboard certificated surgeon (Figure 2). Detailed patient characteristics by surgeon‐certified status are shown in Table S2a,b. Surgical mortality with and without board certification showed significant improvement over time. The AOR using the 2014 outcome for nonboard certificated surgeons as a reference is shown in Table 2. Board‐certificated surgeons demonstrated lower surgical mortality rates than nonboard‐certificated surgeons did and have further improved their AOR over the years (Figure 2A and Table 2); it was 0.817 (95% CI: 0.603–1.108, *p* = 0.193) in 2014 and significantly decreased to 0.522 (95% CI: 0.39–0.7, *p* < 0.001) in 2020. In addition, nonboard certified surgeons have also shown AOR improvement over the years: it was 0.925 (95% CI: 0.75–1.141, *p* = 0.467) in 2015 and significantly decreased to 0.67 (95% CI: 0.542–0.829, *p* < 0.001) in 2020. The AOR for the incidence of grade C pancreatic fistula showed a significant decrease from year to year for both board certificated and nonboard certificated surgeons (Figure 2D and Table 2). In contrast, the 30‐day postoperative mortality showed no clear improvement over time in both board certificated and nonboard certificated surgeons (Figure 2B and Table 2). Although some years showed a trend toward a decrease in the AOR of Clavien–Dindo grade IV or higher complications for both board certificated and nonboard certificated surgeons, the improvement was not significant (Figure 2C and Table 2).

### 3.4 Annual Change in Outcomes by Institution Certificated Status

**FIGURE 3:**
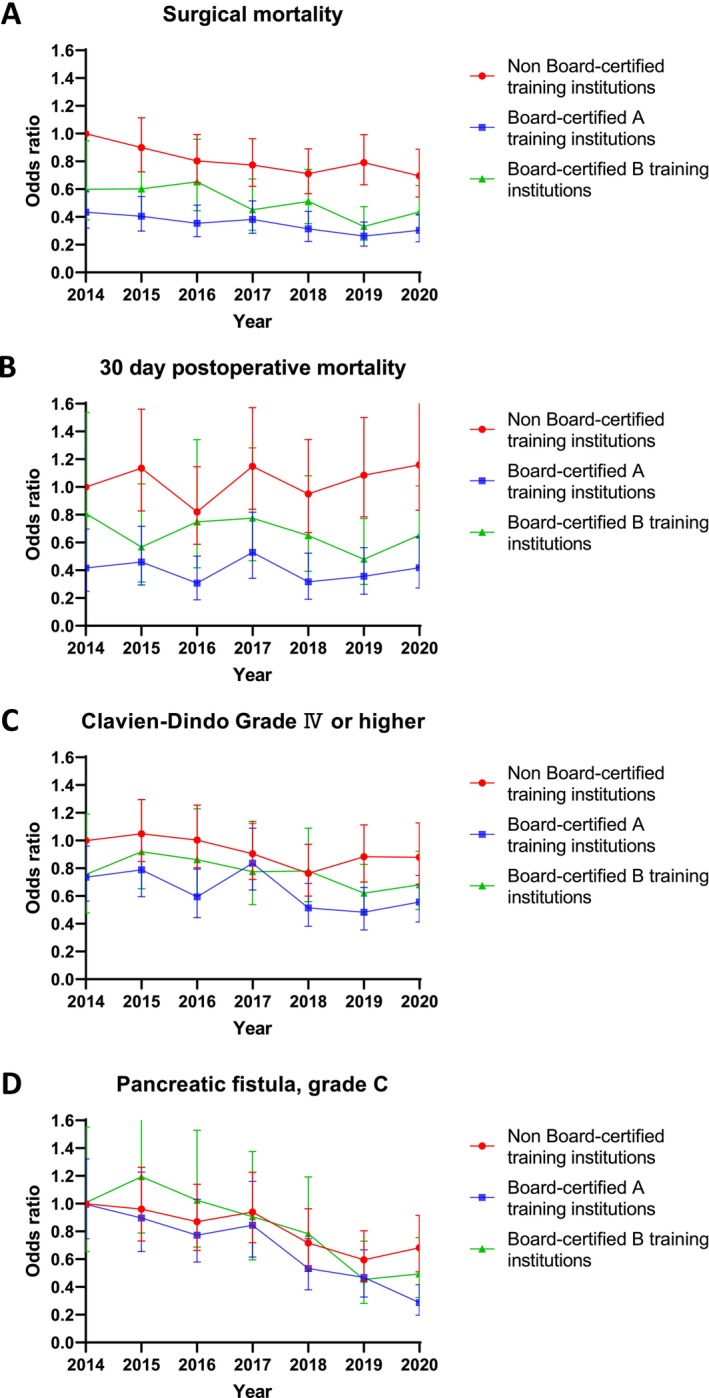
Annual change in odds ratios for surgical mortality by institution status. (A), 30‐day postoperative mortality (B), Clavien‐Dindo ≥ Grade IV (C), and Pancreatic fistula, Grade C (D).

The outcome trends by board‐certified institution status are presented in Figure 3, and detailed patient characteristics are provided in Table S3a–c. Table 2 shows the AOR using 2014 outcomes from nonboard‐certified training institutions as a reference. Significant differences in AORs of surgical mortality were observed between nonboard‐certified and board‐certified A and B institutions as of 2014, with certified institutions demonstrating significantly lower AORs: 434 (95% CI: 0.32–0.587, *p* < 0.001) for institution A and 0.599 (95% CI: 0.38–0.947, *p* = 0.028) for B institutions. Surgical mortality AORs decreased annually across all institutions, with significant reductions observed in 2020:0.695 (95% CI: 0.543–0.888, *p* = 0.004) for nonboard‐certified institutions, 0.436 (95% CI: 0.304–0.626, *p* < 0.001) for board‐certified institutions, and 0.304 (95% CI: 0.221–0.416, *p* < 0.001) for board‐certified institutions. While 30‐day mortality AORs were significantly lower at board‐certified institutions A and B than at nonboard‐certified institutions, nonboard‐certified institutions showed no year‐on‐year improvement (Table 2). The incidence of grade C pancreatic fistula demonstrated a significant annual decrease in all types of training institutions (Figure 3 and Table 2). However, the AORs for Clavien–Dindo grade IV or higher complications varied by institution. Board‐certified institutions A and B consistently maintained lower AORs than nonboard‐certified institutions, albeit with smaller annual improvements. Nonboard‐certified institutions generally lacked year‐on‐year AOR improvements, except in 2018.

## 4 Discussion

This study investigated pancreaticoduodenectomy, a common advanced procedure in hepatobiliary‐pancreatic surgery, using the NCD to analyze temporal changes in odds ratios for surgical mortality, 30‐day postoperative mortality, and major complication rates. Using a previously established risk model as a reference, we observed a substantial decrease in the risk‐adjusted surgical mortality. This study constitutes the first comprehensive analysis globally to demonstrate a trend toward improved surgical outcomes utilizing risk‐adjusted metrics in an extensive longitudinal dataset encompassing the entirety of Japan.

Prior research has reported reductions in postoperative mortality [18] and has emphasized the crucial role of board‐certified surgeons [6]. This study uniquely used changes in AOR based on risk‐adjusted outcomes to precisely evaluate outcome trends. The development of longitudinal case databases across healthcare institutions and the systematic evaluation of medical care performance are crucial for enhancing the quality of care. This report underscores the significant impact of the nationwide database initiative and feedback mechanism of the board certification system in improving surgical quality across the country.

Japan's NCD has validated stepwise board certification systems and has developed risk calculators to tailor surgical treatments to individual needs. It has also enabled international comparisons, enhancing the global surgical quality [10]. Furthermore, the rising complication rates for certain procedures have not led to increased mortality, indicating advancements in perioperative care [19]. Among the Japanese board certification systems aligned with the NCD, JSHBPS board certification is more stringent [6]. This study offers a more comprehensive understanding of the impact of the certification system by examining surgical performance trends over time for both board‐certified and nonboard‐certified surgeons within this framework.

Improving surgical procedures requires accurate data collection. The NCD in Japan, developed in collaboration with the ACS NSQIP, aims to standardize surgical databases for quality enhancement. Both systems collect prospective data using web‐based software to facilitate quality improvement through benchmarking and risk‐adjusted feedback from hospitals [10]. Although ACS‐NSQIP facilities have consistently reduced morbidity and mortality and improved surgical quality [11], some issues have been raised regarding their evaluation. Osborne et al. compared postoperative outcomes and Medicare payments between ACS‐NSQIP and control hospitals and found no significant improvements in outcomes or reductions in Medicare payments for surgical patients associated with participation in the national quality reporting program [12]. Similarly, Etzioni et al., using University HealthSystem Consortium data, found no association between hospital participation in the ACS‐NSQIP and improvements in postoperative outcomes over time [13]. These studies were limited by their reliance on administrative data rather than quality registry data. Nevertheless, they suggested that outcome feedback alone may be insufficient to enhance surgical outcomes. Our findings indicate that the NCD program was not the sole factor in improving surgical results over time, and the implementation of a rigorous board certification system likely played a significant role in this improvement.

Board certification equips surgeons with advanced education, training, and ethical standards, resulting in improved surgical outcomes through enhanced knowledge and adherence to best practices. Studies have indicated that certified surgeons and specialists are consistently associated with lower surgical mortality rates [20]. In Japan, institutional board certification has been shown to significantly decrease the AOR for surgical mortality following hepatectomy [2] and pancreatectomy [21]. Our research revealed an intriguing trend in the AOR for board‐certified and nonboard‐certified institutions and surgeons. Annual analyses showed that the AOR consistently improved over time, regardless of the surgeon's certification status. However, the variation in AOR was more significant for institutional certification than for surgeon certification, particularly for survival mortality. Board‐certified training institutions have greater surgical expertise and more supervisors and specialists. Postpancreatectomy‐specific complications account for almost half of all in‐hospital deaths following pancreatoduodenectomy [22]. Well‐equipped facilities can effectively manage complications and reduce mortality rates. Substantial improvements in surgical mortality have also been observed in non‐certified institutions, suggesting that institutional certification may not be essential for improved outcomes. This aligns with findings from other studies on ACS‐NSQIP participation [13], highlighting the need for further investigation of the factors driving these improvements.

Although surgical mortality has generally declined, 30‐day mortality rates have not significantly improved. 30‐day mortality is typically affected by patient characteristics that cannot be changed, such as age, existing health conditions, and acute incidents like infections, thromboembolism, and cardiovascular events. For example, a separate study corroborated this lack of progress in 30‐day mortality rates, suggesting that cardiac adverse events are a contributing factor [9]. Cardiovascular adverse events are primarily influenced by preoperative factors, including surgery type, functional status, serum creatinine level, ASA class, and preexisting cardiovascular disease [23]. Research indicates that visceral vasculature‐related events and cardiopulmonary complications are the predominant causes of early in‐hospital mortality, often with short intervals between onset and death [22]. Ineffective cardiac quality improvement initiatives did not significantly reduce AOR, potentially explaining the stagnant 30‐day mortality rate. To reduce 30‐day mortality, more comprehensive preoperative evaluations are necessary to identify high‐risk cases and minimize postoperative interventions. Furthermore, variations in immediate postoperative care and patient comorbidities underscore the need for enhanced early postoperative management. Several studies highlight the significance of enhanced perioperative management, strategies for handling high‐risk patients, and better outcomes for complications in reducing 90‐day postoperative mortality. They propose 90‐day mortality as a valid indicator of improved care quality [24, 25]. Thus, the reduction in surgical mortality observed in this study is linked to better surgical outcomes, signifying an enhancement in surgical outcomes.

The observed reduction in surgical mortality can be attributed to several factors. This study suggests that a decrease in grade C pancreatic fistula plays a significant role in the overall reduction of surgical mortality. Grade C pancreatic fistulas are known to contribute to increased mortality rates following pancreatectomy, thereby negatively affecting survival rates [26]. However, implementing specific surgical strategies may improve the outcomes and reduce the mortality rates associated with this complication [27]. Blumgart anastomosis (BA) for pancreaticojejunostomy, which has recently gained popularity, requires further investigation. While some studies have indicated that BA decreases the occurrence of pancreatic fistulas [28], others suggest that it has no effect on these complications [29]. A comprehensive meta‐analysis found that BA significantly reduces the risk of clinically relevant postoperative pancreatic fistula compared with non‐BA techniques [30] and is considered an effective approach. Additionally, there is a growing trend toward more effective postoperative care, such as prompt removal of drainage tubes [31]. Improvements in surgical techniques and postoperative care may have contributed to the decline in pancreatic fistula. Complications above Clavien–Dindo grade IV decreased over time, albeit less significantly than the improvement in pancreatic fistula. Our findings demonstrate that hospitals with greater surgical experience, particularly those certified by the Society, have lower AORs. This suggests that experienced institutions can make appropriate decisions and effectively manage complications, thereby preventing their escalation. This result aligns with previous reports indicating that national efforts have reduced the failure to rescue, particularly in high‐volume centers [32]. The decrease in AOR, despite the aging patient population, indicates that nationwide efforts in Japan are enhancing surgical safety.

This study had several limitations. First, due to the observational nature of this study, there are limitations in establishing a causal relationship between the NCD/certification system and improved outcomes, thus making it challenging to differentiate the effects of the NCD and certification systems more precisely. Second, this study did not account for changes in surgical indications over time between certified and non‐certified facilities, which may influence mortality rates based on operation complexity. Conversion surgery for advanced pancreatic cancer has become more common, but tumor staging, like TNM classification, was not considered. Future studies should evaluate national trends by incorporating preoperative staging and conducting subgroup analyses to assess TNM classification differences between certified and non‐certified facilities for a more precise comparison of mortality outcomes. Another limitation is the inability to analyze more comprehensive characteristics, such as AOR changes, when a board‐certified surgeon operates at a nonboard‐certified institution. Although this complicates the analysis, it provides valuable insights for future research.

In conclusion, this study showed a significant decrease in surgical mortality for pancreaticoduodenectomy, the most common complex hepatobiliary procedure, after participation in the NCD and the introduction of a certification system for highly advanced hepatobiliary and pancreatic surgery. A key factor in this improvement was the reduction in severe postoperative complications, particularly in Grade C pancreatic fistula. This research highlights the potential for enhancing hepatobiliary and pancreatic surgical care through the creation of a longitudinal, multi‐institutional case database and assessment of medical performance, ultimately leading to an improved quality of care.

## Conflicts of Interest

Hiroyuki Yamamoto is affiliated with the Department of Healthcare Quality Assessment of the University of Tokyo. The department is a social collaboration department supported by grants from the National Clinical Database, Intuitive Surgical Sarl, Johnson and Johnson K.K., and Nipro Co. The other authors declare no conflicts of interest.

## Supporting information


Table S1.

Table S2.

Table S3.

## Data Availability

The data that support the findings of this study are available on request from the corresponding author. The data are not publicly available due to privacy or ethical restrictions.
