# Supplementary material for: Trends in the Outcomes of Advanced Hepatobiliary‐Pancreatic Surgery: The Impact of a Nationwide Clinical Database and Surgeon Certification System
Source: J Hepatobiliary Pancreat Sci. 2025 May 13;32(8):565–77. doi: 10.1002/jhbp.12158 (PMC12380033; doi:10.1002/jhbp.12158)
Supplement: Supplementary file 1 — Table S1. [file JHBP-32-565-s001.docx]

| **Supplemental Table 1 Requirements for application to the JSHBPS board certification system** |
| --- |
| **For recognition as a board-certified training institution (HBP field)** |
| Current status as a board-certified training institution in gastroenterology, qualified by the JSGS |
| Employing at least one board-certified instructor or one board-certified expert surgeon |
| Performing 50 or more high-level HBP surgeries per year (for a board-certified A training institution) |
| Performing 30 or more high-level HBP surgeries per year (for a board-certified B training institution) |
| **For recognition as a board-certified instructor (HBP field)** |
| Current status as a board-certified surgeon, qualified by the JSS |
| Current status as a board-certified surgeon in gastroenterology, qualified by the JSGS |
| Experience performing 100 or more high-level HBP surgeries as an operator |
| **For recognition as a board-certified expert surgeon (HBP field)** |
| Current status as a board-certified surgeon, qualified by the JSS |
| Current status as a board-certified surgeon in gastroenterology, qualified by the JSGS |
| Experience performing 50 or more high-level HBP surgeries as an operator during 3-7 years' training at a board-certified training institution (HBP field) |
| Experience performing 10 or more high-level HBP surgeries as a teaching first assistant during 3-7 years' training at a board-certified training institution |
| Video review |
| **High-level HBP surgeries** |
| Right or left hepatic trisegmentectomy |
| Right or extended right or left or extended left hepatectomy |
| Central hepatic bisectionectomy |
| Hepatic sectionectomy (except lateral sectionectomy) |
| Hepatic segmentectomy (S1, S2, S3, S5, S6, S7, S8) |
| Recipient liver transplantation surgery |
| Donor liver transplantation surgery |
| Hepatectomy (S4a + S5 resection or hemihepatectomy) with extrahepatic bile duct resection |
| Extrahepatic bile duct resection for congenital biliary dilatation |
| Hepato-pancreatectomy |
| Total pancreatectomy (including completion pancreatectomy) |
| Pancreatoduodenectomy |
| Distal pancreatectomy with lymph node dissection |
| Spleen-preserving distal pancreatectomy |
| Middle pancreatectomy |
| Duodenum-preserving pancreas head resection |
| Pancreas-sparing duodenectomy |
| Ventral pancreatectomy |
| Inferior pancreas head resection |
| Beger's operation |
| Frey's operation |
| Recipient pancreas transplantation surgery |
| Donor pancreas transplantation surgery |
|  |
| JSHBPS: Japanese Society of Hepato-Biliary-Pancreatic Surgery |
| HBP: hepatobiliary pancreatic |
| JSGS: Japanese Society of Gastroenterological Surgery |
| JSS: Japanese Surgical Society |
