# Supplementary material for: Trends in the Outcomes of Advanced Hepatobiliary‐Pancreatic Surgery: The Impact of a Nationwide Clinical Database and Surgeon Certification System
Source: J Hepatobiliary Pancreat Sci. 2025 May 13;32(8):565–77. doi: 10.1002/jhbp.12158 (PMC12380033; doi:10.1002/jhbp.12158)
Supplement: Supplementary file 2 — Table S2. [file JHBP-32-565-s003.zip › JHBP12158-sup-0003-TableS2b.docx]

| **Supplemental Table 2b**  **Patient characteristics: surgery performed by non-board certificated surgeon** | | | | | | | | |
| --- | --- | --- | --- | --- | --- | --- | --- | --- |
|  |  | 2014 | 2015 | 2016 | 2017 | 2018 | 2019 | 2020 |
|  |  | N=7,193 | N=7,177 | N=7,530 | N=7,919 | N=7,988 | N=8,078 | N=8,192 |
| Age (Years) | -59 | 918 (12.8%) | 946 (13.2%) | 974 (12.9%) | 981 (12.4%) | 980 (12.3%) | 989 (12.2%) | 949 (11.6%) |
|  | 60-64 | 876 (12.2%) | 821 (11.4%) | 777 (10.3%) | 731 (9.2%) | 721 (9.0%) | 752 (9.3%) | 649 (7.9%) |
|  | 65-69 | 1,413 (19.6%) | 1,433 (20.0%) | 1,552 (20.6%) | 1,644 (20.8%) | 1,502 (18.8%) | 1,365 (16.9%) | 1,226 (15.0%) |
|  | 70-74 | 1,636 (22.7%) | 1,668 (23.2%) | 1,630 (21.6%) | 1,712 (21.6%) | 1,750 (21.9%) | 1,846 (22.9%) | 2,018 (24.6%) |
|  | 75-79 | 1,451 (20.2%) | 1,427 (19.9%) | 1,613 (21.4%) | 1,768 (22.3%) | 1,828 (22.9%) | 1,831 (22.7%) | 1,980 (24.2%) |
|  | 80- | 899 (12.5%) | 882 (12.3%) | 984 (13.1%) | 1,083 (13.7%) | 1,207 (15.1%) | 1,295 (16.0%) | 1,370 (16.7%) |
| Male |  | 4,405 (61.2%) | 4,371 (60.9%) | 4,611 (61.2%) | 4,863 (61.4%) | 4,846 (60.7%) | 4,926 (61.0%) | 4,988 (60.9%) |
| COPD |  | 233 (3.2%) | 239 (3.3%) | 294 (3.9%) | 305 (3.9%) | 261 (3.3%) | 290 (3.6%) | 279 (3.4%) |
| Bleeding disorder |  | 261 (3.6%) | 262 (3.7%) | 296 (3.9%) | 348 (4.4%) | 230 (2.9%) | 246 (3.0%) | 277 (3.4%) |
| ASA class (grade 3,4, and 5) |  | 788 (11.0%) | 819 (11.4%) | 997 (13.2%) | 1,009 (12.7%) | 1,099 (13.8%) | 1,160 (14.4%) | 1,317 (16.1%) |
| ASA class (grade 4 and 5) |  | 16 (0.2%) | 21 (0.3%) | 20 (0.3%) | 16 (0.2%) | 23 (0.3%) | 15 (0.2%) | 22 (0.3%) |
| ADL within 30 days before surgery (Partially/totally dependent) |  | 224 (3.1%) | 208 (2.9%) | 237 (3.1%) | 219 (2.8%) | 201 (2.5%) | 195 (2.4%) | 199 (2.4%) |
| BMI >25 |  | 1,174 (16.3%) | 1,182 (16.5%) | 1,223 (16.2%) | 1,370 (17.3%) | 1,359 (17.0%) | 1,492 (18.5%) | 1,488 (18.2%) |
| Weight loss > 10% |  | 456 (6.3%) | 424 (5.9%) | 423 (5.6%) | 448 (5.7%) | 400 (5.0%) | 372 (4.6%) | 384 (4.7%) |
| Brinkman index >400 |  | 1,722 (23.9%) | 1,778 (24.8%) | 1,945 (25.8%) | 2,066 (26.1%) | 2,048 (25.6%) | 2,138 (26.5%) | 2,206 (26.9%) |
| Brinkman index >600 |  | 1,722 (23.9%) | 1,778 (24.8%) | 1,945 (25.8%) | 2,066 (26.1%) | 2,048 (25.6%) | 2,138 (26.5%) | 2,206 (26.9%) |
| Respiratory distress (Within 30 days before surgery) |  | 59 (0.8%) | 70 (1.0%) | 85 (1.1%) | 69 (0.9%) | 54 (0.7%) | 69 (0.9%) | 73 (0.9%) |
| Angina (Within 30 days before surgery) |  | 68 (0.9%) | 85 (1.2%) | 78 (1.0%) | 76 (1.0%) | 90 (1.1%) | 70 (0.9%) | 96 (1.2%) |
| Myocardial infarction (Within 6 months before surgery) |  | 23 (0.3%) | 23 (0.3%) | 17 (0.2%) | 27 (0.3%) | 31 (0.4%) | 19 (0.2%) | 28 (0.3%) |
| Arterial occlusive disease |  | 28 (0.4%) | 18 (0.3%) | 31 (0.4%) | 24 (0.3%) | 28 (0.4%) | 41 (0.5%) | 27 (0.3%) |
| Previous Cerebrovascular disease |  | 220 (3.1%) | 203 (2.8%) | 194 (2.6%) | 291 (3.7%) | 318 (4.0%) | 385 (4.8%) | 343 (4.2%) |
| Ascites without control |  | 66 (0.9%) | 74 (1.0%) | 80 (1.1%) | 82 (1.0%) | 77 (1.0%) | 80 (1.0%) | 71 (0.9%) |
| WBC count >11,000/μl |  | 131 (1.8%) | 142 (2.0%) | 163 (2.2%) | 180 (2.3%) | 170 (2.1%) | 166 (2.1%) | 197 (2.4%) |
| Hemoglobin levels <7g/dl |  | 19 (0.3%) | 12 (0.2%) | 13 (0.2%) | 17 (0.2%) | 24 (0.3%) | 14 (0.2%) | 18 (0.2%) |
| Hematocrit (>48%, male >42%, female) |  | 82 (1.1%) | 101 (1.4%) | 113 (1.5%) | 119 (1.5%) | 138 (1.7%) | 137 (1.7%) | 147 (1.8%) |
| Platelet count <80,000/μl |  | 29 (0.4%) | 22 (0.3%) | 30 (0.4%) | 34 (0.4%) | 28 (0.4%) | 29 (0.4%) | 38 (0.5%) |
| Platelet count <120,000/μl |  | 193 (2.7%) | 213 (3.0%) | 195 (2.6%) | 225 (2.8%) | 194 (2.4%) | 218 (2.7%) | 190 (2.3%) |
| Serum urea nitrogen levels <8mg/dl |  | 386 (5.4%) | 362 (5.0%) | 424 (5.6%) | 420 (5.3%) | 390 (4.9%) | 384 (4.8%) | 370 (4.5%) |
| Serum creatinine levels >2mg/dl |  | 84 (1.2%) | 72 (1.0%) | 87 (1.2%) | 87 (1.1%) | 97 (1.2%) | 79 (1.0%) | 106 (1.3%) |
| Serum creatinine levels >3mg/dl |  | 54 (0.8%) | 46 (0.6%) | 59 (0.8%) | 57 (0.7%) | 68 (0.9%) | 55 (0.7%) | 77 (0.9%) |
| Serum albumin levels <2.5 g/dl |  | 143 (2.0%) | 129 (1.8%) | 160 (2.1%) | 190 (2.4%) | 180 (2.3%) | 177 (2.2%) | 166 (2.0%) |
| Serum sodium level >146mEq/L |  | 27 (0.4%) | 20 (0.3%) | 14 (0.2%) | 18 (0.2%) | 37 (0.5%) | 29 (0.4%) | 37 (0.5%) |
| Serum CRP levels >1.0 mg/dl |  | 1,215 (16.9%) | 1,195 (16.7%) | 1,202 (16.0%) | 1,378 (17.4%) | 1,296 (16.2%) | 1,291 (16.0%) | 1,313 (16.0%) |
| PT-INR >1.1 |  | 804 (11.2%) | 745 (10.4%) | 827 (11.0%) | 811 (10.2%) | 652 (8.2%) | 678 (8.4%) | 720 (8.8%) |
| PT-INR >1.25 |  | 244 (3.4%) | 232 (3.2%) | 192 (2.5%) | 247 (3.1%) | 199 (2.5%) | 201 (2.5%) | 218 (2.7%) |
| APTT >40 sec |  | 289 (4.0%) | 289 (4.0%) | 379 (5.0%) | 317 (4.0%) | 275 (3.4%) | 222 (2.7%) | 246 (3.0%) |
| Duodenal cancer |  | 242 (3.4%) | 275 (3.8%) | 257 (3.4%) | 303 (3.8%) | 305 (3.8%) | 291 (3.6%) | 307 (3.7%) |
| Perihilar bile duct carcinoma |  | 188 (2.6%) | 162 (2.3%) | 176 (2.3%) | 148 (1.9%) | 136 (1.7%) | 133 (1.6%) | 109 (1.3%) |
| Extrahepatic bile duct carcinoma |  | 1,509 (21.0%) | 1,506 (21.0%) | 1,576 (20.9%) | 1,658 (20.9%) | 1,610 (20.2%) | 1,598 (19.8%) | 1,657 (20.2%) |
| Gallbladder cancer |  | 65 (0.9%) | 59 (0.8%) | 66 (0.9%) | 68 (0.9%) | 56 (0.7%) | 61 (0.8%) | 64 (0.8%) |
| Ampulla of Vater carcinoma |  | 924 (12.8%) | 924 (12.9%) | 920 (12.2%) | 956 (12.1%) | 909 (11.4%) | 947 (11.7%) | 908 (11.1%) |
| Multiple metastatic tumor |  | 29 (0.4%) | 25 (0.3%) | 32 (0.4%) | 44 (0.6%) | 25 (0.3%) | 19 (0.2%) | 21 (0.3%) |
| Emergency operation |  | 57 (0.8%) | 48 (0.7%) | 46 (0.6%) | 43 (0.5%) | 49 (0.6%) | 45 (0.6%) | 39 (0.5%) |
| Intraoperative estimated blood loss (ml) | Median (IQR) | 754 (450-1232) | 750 (446-1235) | 710 (420-1174) | 676 (400-1124) | 620 (360-1030) | 600 (340-1020) | 580 (332-980) |
| Operation time (min) | Median (IQR) | 456 (380-545) | 457 (380-541) | 460 (385-545) | 459 (383-545) | 458 (381-547) | 459 (385-542) | 455 (382-538) |
| Vascular reconstruction |  | 708 (9.8%) | 719 (10.0%) | 793 (10.5%) | 703 (8.9%) | 804 (10.1%) | 779 (9.6%) | 909 (11.1%) |
| Length of hospital stay (Days) | Median (IQR) | 30 (21-43) | 29 (20-43) | 29 (19-41) | 28 (19-41) | 28 (19-40) | 27 (19-39) | 26 (18-38) |
| Observed surgical mortality |  | 197 (2.7%) | 182 (2.5%) | 171 (2.3%) | 173 (2.2%) | 157 (2.0%) | 157 (1.9%) | 168 (2.1%) |
| 30-day mortality |  | 83 (1.2%) | 90 (1.3%) | 69 (0.9%) | 104 (1.3%) | 81 (1.0%) | 86 (1.1%) | 107 (1.3%) |
| Clavien-dindo grade IV or higher |  | 186 (2.6%) | 214 (3.0%) | 199 (2.6%) | 215 (2.7%) | 169 (2.1%) | 177 (2.2%) | 203 (2.5%) |
| Pancreatic fistula, grade C |  | 167 (2.3%) | 168 (2.3%) | 160 (2.1%) | 182 (2.3%) | 133 (1.7%) | 110 (1.4%) | 106 (1.3%) |
