# Supplementary material for: Trends in the Outcomes of Advanced Hepatobiliary‐Pancreatic Surgery: The Impact of a Nationwide Clinical Database and Surgeon Certification System
Source: J Hepatobiliary Pancreat Sci. 2025 May 13;32(8):565–77. doi: 10.1002/jhbp.12158 (PMC12380033; doi:10.1002/jhbp.12158)
Supplement: Supplementary file 2 — Table S2. [file JHBP-32-565-s003.zip › JHBP12158-sup-0002-TableS2a.docx]

| **Supplemental Table 2a**  **Patient characteristics: surgery performed by board certificated surgeon** | | | | | | | | |
| --- | --- | --- | --- | --- | --- | --- | --- | --- |
|  |  | 2014 | 2015 | 2016 | 2017 | 2018 | 2019 | 2020 |
|  |  | N=3,206 | N=3,400 | N=3,495 | N=3,663 | N=3,637 | N=3,734 | N=3,759 |
| Age (Years) | -59 | 536 (16.7%) | 560 (16.5%) | 590 (16.9%) | 613 (16.7%) | 569 (15.6%) | 564 (15.1%) | 595 (15.8%) |
|  | 60-64 | 436 (13.6%) | 414 (12.2%) | 363 (10.4%) | 404 (11.0%) | 336 (9.2%) | 322 (8.6%) | 367 (9.8%) |
|  | 65-69 | 629 (19.6%) | 679 (20.0%) | 719 (20.6%) | 715 (19.5%) | 689 (18.9%) | 688 (18.4%) | 611 (16.3%) |
|  | 70-74 | 711 (22.2%) | 751 (22.1%) | 773 (22.1%) | 702 (19.2%) | 829 (22.8%) | 827 (22.1%) | 859 (22.9%) |
|  | 75-79 | 563 (17.6%) | 612 (18.0%) | 630 (18.0%) | 734 (20.0%) | 729 (20.0%) | 781 (20.9%) | 783 (20.8%) |
|  | 80- | 331 (10.3%) | 384 (11.3%) | 420 (12.0%) | 495 (13.5%) | 485 (13.3%) | 552 (14.8%) | 544 (14.5%) |
| Male |  | 1,931 (60.2%) | 2,052 (60.4%) | 2,130 (60.9%) | 2,211 (60.4%) | 2,168 (59.6%) | 2,244 (60.1%) | 2,251 (59.9%) |
| COPD |  | 129 (4.0%) | 145 (4.3%) | 149 (4.3%) | 111 (3.0%) | 107 (2.9%) | 125 (3.3%) | 124 (3.3%) |
| Bleeding disorder |  | 114 (3.6%) | 149 (4.4%) | 121 (3.5%) | 163 (4.4%) | 108 (3.0%) | 98 (2.6%) | 109 (2.9%) |
| ASA class (grade 3,4, and 5) |  | 352 (11.0%) | 384 (11.3%) | 412 (11.8%) | 434 (11.8%) | 499 (13.7%) | 541 (14.5%) | 530 (14.1%) |
| ASA class (grade 4 and 5) |  | 7 　(0.2%) | 11 (0.3%) | 14 (0.4%) | 9　 (0.2%) | 11 (0.3%) | 11 (0.3%) | 16 (0.4%) |
| ADL within 30 days before surgery (Partially/totally dependent) |  | 64 (2.0%) | 72 (2.1%) | 68 (1.9%) | 62 (1.7%) | 62 (1.7%) | 51 (1.4%) | 71 (1.9%) |
| BMI >25 |  | 503 (15.7%) | 530 (15.6%) | 573 (16.4%) | 628 (17.1%) | 666 (18.3%) | 669 (17.9%) | 706 (18.8%) |
| Weight loss > 10% |  | 171 (5.3%) | 200 (5.9%) | 175 (5.0%) | 150 (4.1%) | 172 (4.7%) | 164 (4.4%) | 174 (4.6%) |
| Brinkman index >400 |  | 999 (31.2%) | 1,057 (31.1%) | 1,160 (33.2%) | 1,161 (31.7%) | 1,202 (33.0%) | 1,318 (35.3%) | 1,333 (35.5%) |
| Brinkman index >600 |  | 771 (24.0%) | 817 (24.0%) | 889 (25.4%) | 876 (23.9%) | 901 (24.8%) | 982 (26.3%) | 985 (26.2%) |
| Respiratory distress (Within 30 days before surgery) |  | 29 (0.9%) | 22 (0.6%) | 27 (0.8%) | 28 (0.8%) | 15 (0.4%) | 33 (0.9%) | 37 (1.0%) |
| Angina (Within 30 days before surgery) |  | 45 (1.4%) | 42 (1.2%) | 47 (1.3%) | 55 (1.5%) | 38 (1.0%) | 36 (1.0%) | 32 (0.9%) |
| Myocardial infarction (Within 6 months before surgery) |  | 11 (0.3%) | 13 (0.4%) | 14 (0.4%) | 9　 (0.2%) | 14 (0.4%) | 9　 (0.2%) | 12 (0.3%) |
| Arterial occlusive disease |  | 21 (0.7%) | 13 (0.4%) | 12 (0.3%) | 13 (0.4%) | 15 (0.4%) | 15 (0.4%) | 11 (0.3%) |
| Previous Cerebrovascular disease |  | 101 (3.2%) | 78 (2.3%) | 102 (2.9%) | 125 (3.4%) | 147 (4.0%) | 158 (4.2%) | 161 (4.3%) |
| Ascites without control |  | 41 (1.3%) | 52 (1.5%) | 43 (1.2%) | 32 (0.9%) | 35 (1.0%) | 22 (0.6%) | 47 (1.3%) |
| WBC count >11,000/μl |  | 77 (2.4%) | 80 (2.4%) | 64 (1.8%) | 55 (1.5%) | 77 (2.1%) | 60 (1.6%) | 81 (2.2%) |
| Hemoglobin levels <7g/dl |  | 14 (0.4%) | 8　 (0.2%) | 8　 (0.2%) | 14 (0.4%) | 8　 (0.2%) | 9　 (0.2%) | 10 (0.3%) |
| Hematocrit (>48%, male >42%, female) |  | 25 (0.8%) | 38 (1.1%) | 46 (1.3%) | 61 (1.7%) | 48 (1.3%) | 57 (1.5%) | 69 (1.8%) |
| Platelet count <80,000/μl |  | 14 (0.4%) | 13 (0.4%) | 14 (0.4%) | 17 (0.5%) | 16 (0.4%) | 19 (0.5%) | 11 (0.3%) |
| Platelet count <120,000/μl |  | 95 (3.0%) | 114 (3.4%) | 113 (3.2%) | 109 (3.0%) | 109 (3.0%) | 104 (2.8%) | 132 (3.5%) |
| Serum urea nitrogen levels <8mg/dl |  | 141 (4.4%) | 191 (5.6%) | 184 (5.3%) | 145 (4.0%) | 153 (4.2%) | 158 (4.2%) | 165 (4.4%) |
| Serum creatinine levels >2mg/dl |  | 35 (1.1%) | 35 (1.0%) | 29 (0.8%) | 40 (1.1%) | 47 (1.3%) | 61 (1.6%) | 48 (1.3%) |
| Serum creatinine levels >3mg/dl |  | 28 (0.9%) | 23 (0.7%) | 22 (0.6%) | 30 (0.8%) | 29 (0.8%) | 36 (1.0%) | 35 (0.9%) |
| Serum albumin levels <2.5 g/dl |  | 66 (2.1%) | 60 (1.8%) | 57 (1.6%) | 73 (2.0%) | 55 (1.5%) | 66 (1.8%) | 53 (1.4%) |
| Serum sodium level >146mEq/L |  | 18 (0.6%) | 14 (0.4%) | 14 (0.4%) | 10 (0.3%) | 8　 (0.2%) | 16 (0.4%) | 18 (0.5%) |
| Serum CRP levels >1.0 mg/dl |  | 526 (16.4%) | 550 (16.2%) | 491 (14.0%) | 551 (15.0%) | 509 (14.0%) | 557 (14.9%) | 527 (14.0%) |
| PT-INR >1.1 |  | 357 (11.1%) | 370 (10.9%) | 372 (10.6%) | 343 (9.4%) | 290 (8.0%) | 257 (6.9%) | 280 (7.4%) |
| PT-INR >1.25 |  | 105 (3.3%) | 115 (3.4%) | 93 (2.7%) | 84 (2.3%) | 104 (2.9%) | 80 (2.1%) | 86 (2.3%) |
| APTT >40 sec |  | 114 (3.6%) | 153 (4.5%) | 151 (4.3%) | 133 (3.6%) | 114 (3.1%) | 119 (3.2%) | 92 (2.4%) |
| Duodenal cancer |  | 102 (3.2%) | 106 (3.1%) | 105 (3.0%) | 96 (2.6%) | 82 (2.3%) | 111 (3.0%) | 93 (2.5%) |
| Perihilar bile duct carcinoma |  | 73 (2.3%) | 86 (2.5%) | 85 (2.4%) | 93 (2.5%) | 75 (2.1%) | 55 (1.5%) | 45 (1.2%) |
| Extrahepatic bile duct carcinoma |  | 580 (18.1%) | 577 (17.0%) | 599 (17.1%) | 652 (17.8%) | 607 (16.7%) | 602 (16.1%) | 585 (15.6%) |
| Gallbladder cancer |  | 39 (1.2%) | 38 (1.1%) | 27 (0.8%) | 42 (1.1%) | 38 (1.0%) | 18 (0.5%) | 28 (0.7%) |
| Ampulla of Vater carcinoma |  | 303 (9.5%) | 299 (8.8%) | 329 (9.4%) | 319 (8.7%) | 316 (8.7%) | 309 (8.3%) | 325 (8.6%) |
| Multiple metastatic tumor |  | 12 (0.4%) | 15 (0.4%) | 14 (0.4%) | 13 (0.4%) | 11 (0.3%) | 12 (0.3%) | 17 (0.5%) |
| Emergency operation |  | 22 (0.7%) | 35 (1.0%) | 18 (0.5%) | 27 (0.7%) | 38 (1.0%) | 20 (0.5%) | 31 (0.8%) |
| Intraoperative estimated blood loss (ml) | Median (IQR) | 683.5 (390-1160) | 650 (375-1103.5) | 630 (367-1060) | 590 (320-990) | 543 (290-942) | 501 (270-895) | 515 (270-910) |
| Operation time (min) | Median (IQR) | 457 (371-548) | 454 (371-550) | 459 (376-554) | 442 (360-542) | 446 (365-537) | 435 (355-531) | 446 (365-542) |
| Vascular reconstruction |  | 583 (18.2%) | 613 (18.0%) | 636 (18.2%) | 638 (17.4%) | 623 (17.1%) | 588 (15.7%) | 672 (17.9%) |
| Length of hospital stay (Days) | Median (IQR) | 28 (19-39) | 27 (18-39) | 27 (18-39) | 26 (18-38) | 25 (18-36) | 25 (17-37) | 24 (17-34) |
| Observed surgical mortality |  | 69 (2.2%) | 65 (1.9%) | 70 (2.0%) | 67 (1.8%) | 61 (1.7%) | 58 (1.6%) | 54 (1.4%) |
| 30-day mortality |  | 28 (0.9%) | 30 (0.9%) | 29 (0.8%) | 41 (1.1%) | 30 (0.8%) | 33 (0.9%) | 31 (0.8%) |
| Clavien-dindo grade IV or higher |  | 91 (2.8%) | 87 (2.6%) | 85 (2.4%) | 103 (2.8%) | 79 (2.2%) | 81 (2.2%) | 77 (2.0%) |
| Pancreatic fistula, grade C |  | 85 (2.7%) | 78 (2.3%) | 70 (2.0%) | 76 (2.1%) | 54 (1.5%) | 42 (1.1%) | 37 (1.0%) |
