# Supplementary material for: Trends in the Outcomes of Advanced Hepatobiliary‐Pancreatic Surgery: The Impact of a Nationwide Clinical Database and Surgeon Certification System
Source: J Hepatobiliary Pancreat Sci. 2025 May 13;32(8):565–77. doi: 10.1002/jhbp.12158 (PMC12380033; doi:10.1002/jhbp.12158)
Supplement: Supplementary file 3 — Table S3. [file JHBP-32-565-s002.zip › JHBP12158-sup-0005-TableS3c.docx]

| **Supplemental Table 3c**  **Patient characteristics: surgery performed in board-certified training institutions B** | | | | | | | | |
| --- | --- | --- | --- | --- | --- | --- | --- | --- |
|  |  | 2014 | 2015 | 2016 | 2017 | 2018 | 2019 | 2020 |
|  |  | N=1,289 | N=1,371 | N=1,372 | N=1,642 | N=1,936 | N=2,311 | N=2,418 |
| Age (Years) | -59 | 184 (14.3%) | 191 (13.9%) | 191 (13.9%) | 221 (13.5%) | 256 (13.2%) | 277 (12.0%) | 315 (13.0%) |
|  | 60-64 | 171 (13.3%) | 162 (11.8%) | 141 (10.3%) | 163 (9.9%) | 172 (8.9%) | 215 (9.3%) | 214 (8.9%) |
|  | 65-69 | 274 (21.3%) | 267 (19.5%) | 270 (19.7%) | 304 (18.5%) | 360 (18.6%) | 396 (17.1%) | 370 (15.3%) |
|  | 70-74 | 269 (20.9%) | 314 (22.9%) | 302 (22.0%) | 357 (21.7%) | 421 (21.7%) | 525 (22.7%) | 550 (22.7%) |
|  | 75-79 | 223 (17.3%) | 289 (21.1%) | 296 (21.6%) | 391 (23.8%) | 443 (22.9%) | 515 (22.3%) | 575 (23.8%) |
|  | 80- | 168 (13.0%) | 148 (10.8%) | 172 (12.5%) | 206 (12.5%) | 284 (14.7%) | 383 (16.6%) | 394 (16.3%) |
| Male |  | 763 (59.2%) | 838 (61.1%) | 841 (61.3%) | 986 (60.0%) | 1,165 (60.2%) | 1,423 (61.6%) | 1,448 (59.9%) |
| COPD |  | 25 (1.9%) | 40 (2.9%) | 29 (2.1%) | 41 (2.5%) | 56 (2.9%) | 65 (2.8%) | 47 (1.9%) |
| Bleeding disorder |  | 44 (3.4%) | 44 (3.2%) | 56 (4.1%) | 64 (3.9%) | 58 (3.0%) | 59 (2.6%) | 78 (3.2%) |
| ASA class (grade 3,4, and 5) |  | 146 (11.3%) | 157 (11.5%) | 160 (11.7%) | 228 (13.9%) | 279 (14.4%) | 386 (16.7%) | 423 (17.5%) |
| ASA class (grade 4 and 5) |  | 6 (0.5%) | 4 (0.3%) | 2 (0.1%) | 7 (0.4%) | 8 (0.4%) | 3 (0.1%) | 8 (0.3%) |
| ADL within 30 days before surgery (Partially/totally dependent) |  | 26 (2.0%) | 40 (2.9%) | 33 (2.4%) | 41 (2.5%) | 42 (2.2%) | 42 (1.8%) | 58 (2.4%) |
| BMI >25 |  | 226 (17.5%) | 203 (14.8%) | 230 (16.8%) | 292 (17.8%) | 322 (16.6%) | 418 (18.1%) | 450 (18.6%) |
| Weight loss > 10% |  | 75 (5.8%) | 82 (6.0%) | 75 (5.5%) | 72 (4.4%) | 86 (4.4%) | 96 (4.2%) | 108 (4.5%) |
| Brinkman index >400 |  | 415 (32.2%) | 455 (33.2%) | 476 (34.7%) | 559 (34.0%) | 642 (33.2%) | 858 (37.1%) | 874 (36.1%) |
| Brinkman index >600 |  | 320 (24.8%) | 345 (25.2%) | 366 (26.7%) | 428 (26.1%) | 481 (24.8%) | 649 (28.1%) | 671 (27.8%) |
| Respiratory distress (Within 30 days before surgery) |  | 4 (0.3%) | 9 (0.7%) | 7 (0.5%) | 13 (0.8%) | 11 (0.6%) | 16 (0.7%) | 15 (0.6%) |
| Angina (Within 30 days before surgery) |  | 21 (1.6%) | 20 (1.5%) | 12 (0.9%) | 18 (1.1%) | 21 (1.1%) | 18 (0.8%) | 17 (0.7%) |
| Myocardial infarction (Within 6 months before surgery) |  | 7 (0.5%) | 5 (0.4%) | 4 (0.3%) | 4 (0.2%) | 5 (0.3%) | 7 (0.3%) | 12 (0.5%) |
| Arterial occlusive disease |  | 5 (0.4%) | 6 (0.4%) | 10 (0.7%) | 2 (0.1%) | 5 (0.3%) | 10 (0.4%) | 7 (0.3%) |
| Previous Cerebrovascular disease |  | 33 (2.6%) | 32 (2.3%) | 34 (2.5%) | 61 (3.7%) | 74 (3.8%) | 107 (4.6%) | 91 (3.8%) |
| Ascites without control |  | 5 (0.4%) | 13 (0.9%) | 10 (0.7%) | 15 (0.9%) | 16 (0.8%) | 20 (0.9%) | 14 (0.6%) |
| WBC count >11,000/μl |  | 27 (2.1%) | 29 (2.1%) | 26 (1.9%) | 37 (2.3%) | 54 (2.8%) | 50 (2.2%) | 44 (1.8%) |
| Hemoglobin levels <7g/dl |  | 5 (0.4%) | 3 (0.2%) | 0 (0.0%) | 5 (0.3%) | 3 (0.2%) | 3 (0.1%) | 5 (0.2%) |
| Hematocrit (>48%, male >42%, female) |  | 12 (0.9%) | 20 (1.5%) | 22 (1.6%) | 34 (2.1%) | 32 (1.7%) | 30 (1.3%) | 43 (1.8%) |
| Platelet count <80,000/μl |  | 9 (0.7%) | 7 (0.5%) | 3 (0.2%) | 4 (0.2%) | 9 (0.5%) | 9 (0.4%) | 10 (0.4%) |
| Platelet count <120,000/μl |  | 45 (3.5%) | 45 (3.3%) | 44 (3.2%) | 42 (2.6%) | 56 (2.9%) | 61 (2.6%) | 73 (3.0%) |
| Serum urea nitrogen levels <8mg/dl |  | 53 (4.1%) | 52 (3.8%) | 67 (4.9%) | 69 (4.2%) | 94 (4.9%) | 107 (4.6%) | 118 (4.9%) |
| Serum creatinine levels >2mg/dl |  | 14 (1.1%) | 10 (0.7%) | 12 (0.9%) | 14 (0.9%) | 27 (1.4%) | 28 (1.2%) | 25 (1.0%) |
| Serum creatinine levels >3mg/dl |  | 8 (0.6%) | 5 (0.4%) | 9 (0.7%) | 10 (0.6%) | 20 (1.0%) | 20 (0.9%) | 17 (0.7%) |
| Serum albumin levels <2.5 g/dl |  | 28 (2.2%) | 23 (1.7%) | 24 (1.7%) | 46 (2.8%) | 59 (3.0%) | 55 (2.4%) | 43 (1.8%) |
| Serum sodium level >146mEq/L |  | 3 (0.2%) | 2 (0.1%) | 1 (0.1%) | 5 (0.3%) | 10 (0.5%) | 10 (0.4%) | 14 (0.6%) |
| Serum CRP levels >1.0 mg/dl |  | 241 (18.7%) | 221 (16.1%) | 208 (15.2%) | 291 (17.7%) | 290 (15.0%) | 399 (17.3%) | 385 (15.9%) |
| PT-INR >1.1 |  | 117 (9.1%) | 146 (10.6%) | 164 (12.0%) | 178 (10.8%) | 162 (8.4%) | 180 (7.8%) | 220 (9.1%) |
| PT-INR >1.25 |  | 45 (3.5%) | 49 (3.6%) | 36 (2.6%) | 49 (3.0%) | 69 (3.6%) | 56 (2.4%) | 67 (2.8%) |
| APTT >40 sec |  | 58 (4.5%) | 47 (3.4%) | 64 (4.7%) | 64 (3.9%) | 73 (3.8%) | 64 (2.8%) | 69 (2.9%) |
| Duodenal cancer |  | 52 (4.0%) | 46 (3.4%) | 41 (3.0%) | 56 (3.4%) | 59 (3.0%) | 85 (3.7%) | 77 (3.2%) |
| Perihilar bile duct carcinoma |  | 36 (2.8%) | 20 (1.5%) | 38 (2.8%) | 39 (2.4%) | 33 (1.7%) | 35 (1.5%) | 35 (1.4%) |
| Extrahepatic bile duct carcinoma |  | 241 (18.7%) | 261 (19.0%) | 299 (21.8%) | 322 (19.6%) | 380 (19.6%) | 459 (19.9%) | 437 (18.1%) |
| Gallbladder cancer |  | 10 (0.8%) | 12 (0.9%) | 14 (1.0%) | 17 (1.0%) | 15 (0.8%) | 20 (0.9%) | 14 (0.6%) |
| Ampulla of Vater carcinoma |  | 161 (12.5%) | 163 (11.9%) | 160 (11.7%) | 187 (11.4%) | 195 (10.1%) | 235 (10.2%) | 241 (10.0%) |
| Multiple metastatic tumor |  | 3 (0.2%) | 6 (0.4%) | 9 (0.7%) | 4 (0.2%) | 2 (0.1%) | 7 (0.3%) | 7 (0.3%) |
| Emergency operation |  | 10 (0.8%) | 11 (0.8%) | 9 (0.7%) | 5 (0.3%) | 16 (0.8%) | 9 (0.4%) | 12 (0.5%) |
| Intraoperative estimated blood loss (ml) | Median (IQR) | 740 (450-1220) | 700 (412-1172) | 685 (420-1149) | 655.5 (380-1090) | 580 (320-1013.5) | 568 (311-950) | 545 (300-950) |
| Operation time (min) | Median (IQR) | 440 (367-523) | 439 (366-528) | 447 (377.5-527.5) | 443.5 (376-529) | 447 (378-528) | 446 (376-526) | 449.5 (376-536) |
| Vascular reconstruction |  | 163 (12.6%) | 169 (12.3%) | 180 (13.1%) | 190 (11.6%) | 235 (12.1%) | 286 (12.4%) | 365 (15.1%) |
| Length of hospital stay (Days) | Median (IQR) | 27 (20-40) | 27 (19-40) | 26 (19-38) | 27 (19-39) | 26 (18-38) | 26 (19-37) | 25 (18-36) |
| Observed surgical mortality |  | 27 (2.1%) | 28 (2.0%) | 32 (2.3%) | 27 (1.6%) | 36 (1.9%) | 29 (1.3%) | 41 (1.7%) |
| 30-day mortality |  | 15 (1.2%) | 11 (0.8%) | 15 (1.1%) | 19 (1.2%) | 19 (1.0%) | 17 (0.7%) | 24 (1.0%) |
| Clavien-dindo grade IV or higher |  | 30 (2.3%) | 38 (2.8%) | 36 (2.6%) | 41 (2.5%) | 49 (2.5%) | 47 (2.0%) | 53 (2.2%) |
| Pancreatic fistula, grade C |  | 31 (2.4%) | 38 (2.8%) | 35 (2.6%) | 37 (2.3%) | 37 (1.9%) | 26 (1.1%) | 29 (1.2%) |
