# Supplementary material for: Trends in the Outcomes of Advanced Hepatobiliary‐Pancreatic Surgery: The Impact of a Nationwide Clinical Database and Surgeon Certification System
Source: J Hepatobiliary Pancreat Sci. 2025 May 13;32(8):565–77. doi: 10.1002/jhbp.12158 (PMC12380033; doi:10.1002/jhbp.12158)
Supplement: Supplementary file 3 — Table S3. [file JHBP-32-565-s002.zip › JHBP12158-sup-0004-TableS3a.docx]

| **Supplemental Table 3a**  **Patient characteristics: surgery performed in non-board-certified training institutions** | | | | | | | | |
| --- | --- | --- | --- | --- | --- | --- | --- | --- |
|  |  | 2014 | 2015 | 2016 | 2017 | 2018 | 2019 | 2020 |
|  |  | N=4,947 | N=4,862 | N=5,051 | N=5,073 | N=4,819 | N=4,452 | N=4,375 |
| Age (Years) | -59 | 585 (11.8%) | 611 (12.6%) | 597 (11.8%) | 563 (11.1%) | 537 (11.1%) | 483 (10.8%) | 439 (10.0%) |
|  | 60-64 | 581 (11.7%) | 521 (10.7%) | 515 (10.2%) | 481 (9.5%) | 418 (8.7%) | 394 (8.8%) | 338 (7.7%) |
|  | 65-69 | 921 (18.6%) | 934 (19.2%) | 1,015 (20.1%) | 1,059 (20.9%) | 895 (18.6%) | 734 (16.5%) | 651 (14.9%) |
|  | 70-74 | 1,178 (23.8%) | 1,142 (23.5%) | 1,078 (21.3%) | 1,058 (20.9%) | 1,078 (22.4%) | 1,003 (22.5%) | 1,096 (25.1%) |
|  | 75-79 | 1,046 (21.1%) | 986 (20.3%) | 1,126 (22.3%) | 1,139 (22.5%) | 1,099 (22.8%) | 1,053 (23.7%) | 1,051 (24.0%) |
|  | 80- | 636 (12.9%) | 668 (13.7%) | 720 (14.3%) | 773 (15.2%) | 792 (16.4%) | 785 (17.6%) | 800 (18.3%) |
| Male |  | 3,093 (62.5%) | 2,925 (60.2%) | 3,123 (61.8%) | 3,184 (62.8%) | 2,887 (59.9%) | 2,735 (61.4%) | 2,702 (61.8%) |
| COPD |  | 135 (2.7%) | 124 (2.6%) | 148 (2.9%) | 135 (2.7%) | 127 (2.6%) | 134 (3.0%) | 150 (3.4%) |
| Bleeding disorder |  | 196 (4.0%) | 170 (3.5%) | 175 (3.5%) | 225 (4.4%) | 134 (2.8%) | 136 (3.1%) | 122 (2.8%) |
| ASA class (grade 3,4, and 5) |  | 608 (12.3%) | 670 (13.8%) | 760 (15.0%) | 708 (14.0%) | 761 (15.8%) | 702 (15.8%) | 767 (17.5%) |
| ASA class (grade 4 and 5) |  | 12 (0.2%) | 22 (0.5%) | 15 (0.3%) | 12 (0.2%) | 16 (0.3%) | 12 (0.3%) | 16 (0.4%) |
| ADL within 30 days before surgery (Partially/totally dependent) |  | 184 (3.7%) | 176 (3.6%) | 186 (3.7%) | 153 (3.0%) | 122 (2.5%) | 140 (3.1%) | 124 (2.8%) |
| BMI >25 |  | 184 (3.7%) | 176 (3.6%) | 186 (3.7%) | 153 (3.0%) | 122 (2.5%) | 140 (3.1%) | 124 (2.8%) |
| Weight loss > 10% |  | 350 (7.1%) | 317 (6.5%) | 297 (5.9%) | 280 (5.5%) | 257 (5.3%) | 219 (4.9%) | 231 (5.3%) |
| Brinkman index >400 |  | 1,439 (29.1%) | 1,485 (30.5%) | 1,610 (31.9%) | 1,579 (31.1%) | 1,550 (32.2%) | 1,481 (33.3%) | 1,514 (34.6%) |
| Brinkman index >600 |  | 1,122 (22.7%) | 1,155 (23.8%) | 1,229 (24.3%) | 1,220 (24.0%) | 1,177 (24.4%) | 1,129 (25.4%) | 1,138 (26.0%) |
| Respiratory distress (Within 30 days before surgery) |  | 55 (1.1%) | 51 (1.0%) | 68 (1.3%) | 45 (0.9%) | 28 (0.6%) | 48 (1.1%) | 52 (1.2%) |
| Angina (Within 30 days before surgery) |  | 50 (1.0%) | 58 (1.2%) | 51 (1.0%) | 47 (0.9%) | 60 (1.2%) | 37 (0.8%) | 53 (1.2%) |
| Myocardial infarction (Within 6 months before surgery) |  | 11 (0.2%) | 18 (0.4%) | 12 (0.2%) | 21 (0.4%) | 22 (0.5%) | 8 (0.2%) | 13 (0.3%) |
| Arterial occlusive disease |  | 19 (0.4%) | 11 (0.2%) | 18 (0.4%) | 22 (0.4%) | 19 (0.4%) | 22 (0.5%) | 13 (0.3%) |
| Previous Cerebrovascular disease |  | 168 (3.4%) | 146 (3.0%) | 142 (2.8%) | 193 (3.8%) | 199 (4.1%) | 238 (5.3%) | 203 (4.6%) |
| Ascites without control |  | 55 (1.1%) | 64 (1.3%) | 73 (1.4%) | 58 (1.1%) | 59 (1.2%) | 53 (1.2%) | 63 (1.4%) |
| WBC count >11,000/μl |  | 121 (2.4%) | 109 (2.2%) | 123 (2.4%) | 121 (2.4%) | 111 (2.3%) | 101 (2.3%) | 120 (2.7%) |
| Hemoglobin levels <7g/dl |  | 14 (0.3%) | 10 (0.2%) | 10 (0.2%) | 13 (0.3%) | 20 (0.4%) | 11 (0.2%) | 9 (0.2%) |
| Hematocrit (>48%, male >42%, female) |  | 51 (1.0%) | 44 (0.9%) | 63 (1.2%) | 70 (1.4%) | 77 (1.6%) | 61 (1.4%) | 76 (1.7%) |
| Platelet count <80,000/μl |  | 21 (0.4%) | 12 (0.2%) | 20 (0.4%) | 26 (0.5%) | 15 (0.3%) | 16 (0.4%) | 18 (0.4%) |
| Platelet count <120,000/μl |  | 125 (2.5%) | 124 (2.6%) | 120 (2.4%) | 148 (2.9%) | 121 (2.5%) | 122 (2.7%) | 105 (2.4%) |
| Serum urea nitrogen levels <8mg/dl |  | 303 (6.1%) | 315 (6.5%) | 320 (6.3%) | 291 (5.7%) | 262 (5.4%) | 252 (5.7%) | 235 (5.4%) |
| Serum creatinine levels >2mg/dl |  | 61 (1.2%) | 48 (1.0%) | 55 (1.1%) | 66 (1.3%) | 52 (1.1%) | 49 (1.1%) | 50 (1.1%) |
| Serum creatinine levels >3mg/dl |  | 43 (0.9%) | 30 (0.6%) | 40 (0.8%) | 45 (0.9%) | 38 (0.8%) | 33 (0.7%) | 39 (0.9%) |
| Serum albumin levels <2.5 g/dl |  | 126 (2.5%) | 103 (2.1%) | 121 (2.4%) | 144 (2.8%) | 121 (2.5%) | 110 (2.5%) | 107 (2.4%) |
| Serum sodium level >146mEq/L |  | 23 (0.5%) | 17 (0.3%) | 12 (0.2%) | 12 (0.2%) | 17 (0.4%) | 15 (0.3%) | 15 (0.3%) |
| Serum CRP levels >1.0 mg/dl |  | 935 (18.9%) | 920 (18.9%) | 895 (17.7%) | 972 (19.2%) | 879 (18.2%) | 818 (18.4%) | 776 (17.7%) |
| PT-INR >1.1 |  | 646 (13.1%) | 581 (11.9%) | 555 (11.0%) | 545 (10.7%) | 443 (9.2%) | 424 (9.5%) | 393 (9.0%) |
| PT-INR >1.25 |  | 192 (3.9%) | 191 (3.9%) | 148 (2.9%) | 183 (3.6%) | 124 (2.6%) | 139 (3.1%) | 119 (2.7%) |
| APTT >40 sec |  | 192 (3.9%) | 225 (4.6%) | 249 (4.9%) | 226 (4.5%) | 178 (3.7%) | 137 (3.1%) | 122 (2.8%) |
| Duodenal cancer |  | 174 (3.5%) | 202 (4.2%) | 182 (3.6%) | 191 (3.8%) | 186 (3.9%) | 161 (3.6%) | 153 (3.5%) |
| Perihilar bile duct carcinoma |  | 143 (2.9%) | 128 (2.6%) | 135 (2.7%) | 115 (2.3%) | 92 (1.9%) | 92 (2.1%) | 74 (1.7%) |
| Extrahepatic bile duct carcinoma |  | 1,148 (23.2%) | 1,094 (22.5%) | 1,117 (22.1%) | 1,151 (22.7%) | 1,052 (21.8%) | 951 (21.4%) | 987 (22.6%) |
| Gallbladder cancer |  | 54 (1.1%) | 53 (1.1%) | 52 (1.0%) | 48 (0.9%) | 40 (0.8%) | 34 (0.8%) | 46 (1.1%) |
| Ampulla of Vater carcinoma |  | 611 (12.4%) | 622 (12.8%) | 589 (11.7%) | 609 (12.0%) | 548 (11.4%) | 512 (11.5%) | 487 (11.1%) |
| Multiple metastatic tumor |  | 26 (0.5%) | 19 (0.4%) | 24 (0.5%) | 30 (0.6%) | 18 (0.4%) | 17 (0.4%) | 16 (0.4%) |
| Emergency operation |  | 43 (0.9%) | 42 (0.9%) | 40 (0.8%) | 36 (0.7%) | 24 (0.5%) | 28 (0.6%) | 29 (0.7%) |
| Intraoperative estimated blood loss (ml) | Median (IQR) | 800 (494-1300) | 800 (486-1300) | 770 (460-1255) | 739 (450-1202) | 690 (400-1131) | 674 (390-1139.5) | 650 (383-1100) |
| Operation time (min) | Median (IQR) | 458 (380-543) | 456 (378-539) | 460 (384-548) | 458 (382-546) | 458 (378-548) | 462 (382-550) | 457 (380-544) |
| Vascular reconstruction |  | 452 (9.1%) | 411 (8.5%) | 477 (9.4%) | 405 (8.0%) | 422 (8.8%) | 376 (8.4%) | 403 (9.2%) |
| Length of hospital stay (Days) | Median (IQR) | 32 (23-46) | 32 (22-45) | 31 (21-45) | 30 (21-43) | 30 (21-43) | 29 (20-42) | 29 (20-41) |
| Observed surgical mortality |  | 179 (3.6%) | 160 (3.3%) | 153 (3.0%) | 148 (2.9%) | 128 (2.7%) | 138 (3.1%) | 123 (2.8%) |
| 30-day mortality |  | 72 (1.5%) | 81 (1.7%) | 63 (1.2%) | 89 (1.8%) | 69 (1.4%) | 75 (1.7%) | 82 (1.9%) |
| Clavien-dindo grade IV or higher |  | 156 (3.2%) | 160 (3.3%) | 164 (3.2%) | 152 (3.0%) | 118 (2.4%) | 132 (3.0%) | 133 (3.0%) |
| Pancreatic fistula, grade C |  | 127 (2.6%) | 119 (2.4%) | 114 (2.3%) | 126 (2.5%) | 89 (1.8%) | 70 (1.6%) | 80 (1.8%) |
