# Supplementary material for: Trends in the Outcomes of Advanced Hepatobiliary‐Pancreatic Surgery: The Impact of a Nationwide Clinical Database and Surgeon Certification System
Source: J Hepatobiliary Pancreat Sci. 2025 May 13;32(8):565–77. doi: 10.1002/jhbp.12158 (PMC12380033; doi:10.1002/jhbp.12158)
Supplement: Supplementary file 3 — Table S3. [file JHBP-32-565-s002.zip › JHBP12158-sup-0005-TableS3b.docx]

| **Supplemental Table 3b**  **Patient characteristics: surgery performed in board-certified training institutions A** | | | | | | | | |
| --- | --- | --- | --- | --- | --- | --- | --- | --- |
|  |  | 2014 | 2015 | 2016 | 2017 | 2018 | 2019 | 2020 |
|  |  | N=4,163 | N=4,344 | N=4,602 | N=4,867 | N=4,870 | N=5,049 | N=5,158 |
| Age (Years) | -59 | 685 (16.5%) | 704 (16.2%) | 776 (16.9%) | 810 (16.6%) | 756 (15.5%) | 793 (15.7%) | 790 (15.3%) |
|  | 60-64 | 560 (13.5%) | 552 (12.7%) | 484 (10.5%) | 491 (10.1%) | 467 (9.6%) | 465 (9.2%) | 464 (9.0%) |
|  | 65-69 | 847 (20.3%) | 911 (21.0%) | 986 (21.4%) | 996 (20.5%) | 936 (19.2%) | 923 (18.3%) | 816 (15.8%) |
|  | 70-74 | 900 (21.6%) | 963 (22.2%) | 1,023 (22.2%) | 999 (20.5%) | 1,080 (22.2%) | 1,145 (22.7%) | 1,231 (23.9%) |
|  | 75-79 | 745 (17.9%) | 764 (17.6%) | 821 (17.8%) | 972 (20.0%) | 1,015 (20.8%) | 1,044 (20.7%) | 1,137 (22.0%) |
|  | 80- | 426 (10.2%) | 450 (10.4%) | 512 (11.1%) | 599 (12.3%) | 616 (12.6%) | 679 (13.4%) | 720 (14.0%) |
| Male |  | 2,480 (59.6%) | 2,660 (61.2%) | 2,777 (60.3%) | 2,904 (59.7%) | 2,962 (60.8%) | 3,012 (59.7%) | 3,089 (59.9%) |
| COPD |  | 202 (4.9%) | 220 (5.1%) | 266 (5.8%) | 240 (4.9%) | 185 (3.8%) | 216 (4.3%) | 206 (4.0%) |
| Bleeding disorder |  | 135 (3.2%) | 197 (4.5%) | 186 (4.0%) | 222 (4.6%) | 146 (3.0%) | 149 (3.0%) | 186 (3.6%) |
| ASA class (grade 3,4, and 5) |  | 396 (9.5%) | 393 (9.0%) | 507 (11.0%) | 516 (10.6%) | 573 (11.8%) | 625 (12.4%) | 671 (13.0%) |
| ASA class (grade 4 and 5) |  | 5 (0.1%) | 6 (0.1%) | 17 (0.4%) | 6 (0.1%) | 10 (0.2%) | 11 (0.2%) | 14 (0.3%) |
| ADL within 30 days before surgery (Partially/totally dependent) |  | 89 (2.1%) | 79 (1.8%) | 98 (2.1%) | 91 (1.9%) | 109 (2.2%) | 80 (1.6%) | 94 (1.8%) |
| BMI >25 |  | 640 (15.4%) | 690 (15.9%) | 731 (15.9%) | 830 (17.1%) | 886 (18.2%) | 940 (18.6%) | 949 (18.4%) |
| Weight loss > 10% |  | 202 (4.9%) | 225 (5.2%) | 226 (4.9%) | 246 (5.1%) | 229 (4.7%) | 221 (4.4%) | 219 (4.2%) |
| Brinkman index >400 |  | 1,329 (31.9%) | 1,431 (32.9%) | 1,614 (35.1%) | 1,695 (34.8%) | 1,683 (34.6%) | 1,786 (35.4%) | 1,841 (35.7%) |
| Brinkman index >600 |  | 1,051 (25.2%) | 1,095 (25.2%) | 1,239 (26.9%) | 1,294 (26.6%) | 1,291 (26.5%) | 1,342 (26.6%) | 1,382 (26.8%) |
| Respiratory distress (Within 30 days before surgery) |  | 29 (0.7%) | 32 (0.7%) | 37 (0.8%) | 39 (0.8%) | 30 (0.6%) | 38 (0.8%) | 43 (0.8%) |
| Angina (Within 30 days before surgery) |  | 42 (1.0%) | 49 (1.1%) | 62 (1.3%) | 66 (1.4%) | 47 (1.0%) | 51 (1.0%) | 58 (1.1%) |
| Myocardial infarction (Within 6 months before surgery) |  | 16 (0.4%) | 13 (0.3%) | 15 (0.3%) | 11 (0.2%) | 18 (0.4%) | 13 (0.3%) | 15 (0.3%) |
| Arterial occlusive disease |  | 25 (0.6%) | 14 (0.3%) | 15 (0.3%) | 13 (0.3%) | 19 (0.4%) | 24 (0.5%) | 18 (0.3%) |
| Previous Cerebrovascular disease |  | 120 (2.9%) | 103 (2.4%) | 120 (2.6%) | 162 (3.3%) | 192 (3.9%) | 198 (3.9%) | 210 (4.1%) |
| Ascites without control |  | 47 (1.1%) | 49 (1.1%) | 40 (0.9%) | 41 (0.8%) | 37 (0.8%) | 29 (0.6%) | 41 (0.8%) |
| WBC count >11,000/μl |  | 60 (1.4%) | 84 (1.9%) | 78 (1.7%) | 77 (1.6%) | 82 (1.7%) | 75 (1.5%) | 114 (2.2%) |
| Hemoglobin levels <7g/dl |  | 14 (0.3%) | 7 (0.2%) | 11 (0.2%) | 13 (0.3%) | 9 (0.2%) | 9 (0.2%) | 14 (0.3%) |
| Hematocrit (>48%, male >42%, female) |  | 44 (1.1%) | 75 (1.7%) | 74 (1.6%) | 76 (1.6%) | 77 (1.6%) | 103 (2.0%) | 97 (1.9%) |
| Platelet count <80,000/μl |  | 13 (0.3%) | 16 (0.4%) | 21 (0.5%) | 21 (0.4%) | 20 (0.4%) | 23 (0.5%) | 21 (0.4%) |
| Platelet count <120,000/μl |  | 118 (2.8%) | 158 (3.6%) | 144 (3.1%) | 144 (3.0%) | 126 (2.6%) | 139 (2.8%) | 144 (2.8%) |
| Serum urea nitrogen levels <8mg/dl |  | 171 (4.1%) | 186 (4.3%) | 221 (4.8%) | 205 (4.2%) | 187 (3.8%) | 183 (3.6%) | 182 (3.5%) |
| Serum creatinine levels >2mg/dl |  | 44 (1.1%) | 49 (1.1%) | 49 (1.1%) | 47 (1.0%) | 65 (1.3%) | 63 (1.2%) | 79 (1.5%) |
| Serum creatinine levels >3mg/dl |  | 31 (0.7%) | 34 (0.8%) | 32 (0.7%) | 32 (0.7%) | 39 (0.8%) | 38 (0.8%) | 56 (1.1%) |
| Serum albumin levels <2.5 g/dl |  | 55 (1.3%) | 63 (1.5%) | 72 (1.6%) | 73 (1.5%) | 55 (1.1%) | 78 (1.5%) | 69 (1.3%) |
| Serum sodium level >146mEq/L |  | 19 (0.5%) | 15 (0.3%) | 15 (0.3%) | 11 (0.2%) | 18 (0.4%) | 20 (0.4%) | 26 (0.5%) |
| Serum CRP levels >1.0 mg/dl |  | 565 (13.6%) | 604 (13.9%) | 590 (12.8%) | 666 (13.7%) | 636 (13.1%) | 631 (12.5%) | 679 (13.2%) |
| PT-INR >1.1 |  | 398 (9.6%) | 388 (8.9%) | 480 (10.4%) | 431 (8.9%) | 337 (6.9%) | 331 (6.6%) | 387 (7.5%) |
| PT-INR >1.25 |  | 112 (2.7%) | 107 (2.5%) | 101 (2.2%) | 99 (2.0%) | 110 (2.3%) | 86 (1.7%) | 118 (2.3%) |
| APTT >40 sec |  | 153 (3.7%) | 170 (3.9%) | 217 (4.7%) | 160 (3.3%) | 138 (2.8%) | 140 (2.8%) | 147 (2.8%) |
| Duodenal cancer |  | 118 (2.8%) | 133 (3.1%) | 139 (3.0%) | 152 (3.1%) | 142 (2.9%) | 156 (3.1%) | 170 (3.3%) |
| Perihilar bile duct carcinoma |  | 82 (2.0%) | 100 (2.3%) | 88 (1.9%) | 87 (1.8%) | 86 (1.8%) | 61 (1.2%) | 45 (0.9%) |
| Extrahepatic bile duct carcinoma |  | 700 (16.8%) | 728 (16.8%) | 759 (16.5%) | 837 (17.2%) | 785 (16.1%) | 790 (15.6%) | 818 (15.9%) |
| Gallbladder cancer |  | 40 (1.0%) | 32 (0.7%) | 27 (0.6%) | 45 (0.9%) | 39 (0.8%) | 25 (0.5%) | 32 (0.6%) |
| Ampulla of Vater carcinoma |  | 455 (10.9%) | 438 (10.1%) | 500 (10.9%) | 479 (9.8%) | 482 (9.9%) | 509 (10.1%) | 505 (9.8%) |
| Multiple metastatic tumor |  | 12 (0.3%) | 15 (0.3%) | 13 (0.3%) | 23 (0.5%) | 16 (0.3%) | 7 (0.1%) | 15 (0.3%) |
| Emergency operation |  | 26 (0.6%) | 30 (0.7%) | 15 (0.3%) | 29 (0.6%) | 47 (1.0%) | 28 (0.6%) | 29 (0.6%) |
| Intraoperative estimated blood loss (ml) | Median (IQR) | 650 (366-1095) | 633.5 (370-1068.5) | 596 (345-989) | 555 (300-940) | 520 (289-895) | 490 (270-855) | 492 (265-870) |
| Operation time (min) | Median (IQR) | 460 (379-557) | 462 (379.5-556) | 465 (383-555) | 454 (368-548) | 455.5 (373-548) | 445 (367-534) | 450 (374-537) |
| Vascular reconstruction |  | 676 (16.2%) | 752 (17.3%) | 772 (16.8%) | 746 (15.3%) | 770 (15.8%) | 705 (14.0%) | 813 (15.8%) |
| Length of hospital stay (Days) | Median (IQR) | 26 (18-38) | 25 (17-37) | 25 (17-36) | 25 (17-36) | 24 (17-36) | 24 (17-35) | 23 (17-33) |
| Observed surgical mortality |  | 60 (1.4%) | 59 (1.4%) | 56 (1.2%) | 65 (1.3%) | 54 (1.1%) | 48 (1.0%) | 58 (1.1%) |
| 30-day mortality |  | 24 (0.6%) | 28 (0.6%) | 20 (0.4%) | 37 (0.8%) | 23 (0.5%) | 27 (0.5%) | 32 (0.6%) |
| Clavien-dindo grade IV or higher |  | 91 (2.2%) | 103 (2.4%) | 84 (1.8%) | 125 (2.6%) | 81 (1.7%) | 79 (1.6%) | 94 (1.8%) |
| Pancreatic fistula, grade C |  | 94 (2.3%) | 89 (2.0%) | 81 (1.8%) | 95 (2.0%) | 61 (1.3%) | 56 (1.1%) | 34 (0.7%) |
